# Ethical and legal challenges with IoT in home digital twins

**DOI:** 10.1016/j.mex.2025.103409

**Published:** 2025-05-30

**Authors:** D. Dhinakaran, S. Edwin Raja, A. Ramathilagam, G. Vennila, A. Alagulakshmi

**Affiliations:** a Department of Computer Science and Engineering, Vel Tech Rangarajan Dr. Sagunthala R&D Institute of Science and Technology, Chennai, India; b Department of Computer Science and Engineering, P.S.R Engineering College, Sivakasi, India; c Department of Artificial Intelligence and Machine Learning, School of Computing, Mohan Babu University, Tirupati, India; d Department of Information Technology, Ramco Institute of Technology, Rajapalayam, India

**Keywords:** Internet of Things, Home digital twins, Privacy, Data security, Trust, Cybersecurity, Ethico-Legal IoT Compliance Framework (ELICF)

## Abstract

Home Digital Twins represent a transformative application of IoT in the home environment, turning conventional living spaces into intelligent ecosystems. This paper explores the ethical and legal challenges associated with these technologies, focusing on critical issues such as privacy, data security, and accountability. The study integrates real-world case studies of privacy controversies and cybersecurity breaches to illustrate potential vulnerabilities in IoT-enabled systems. Furthermore, it examines the complexities of regulatory compliance, including cross-border data flows and liability concerns in the event of system failures, with a focus on frameworks such as GDPR, CCPA, and India’s Digital Personal Data Protection Bill. The methodology includes an in-depth analysis of existing legal frameworks, industry best practices, and technical mitigation strategies to propose actionable guidelines for addressing these challenges. Key findings emphasize the necessity of robust legal frameworks, user-centered design principles, and transparent data practices to foster trust and security in IoT systems. By advocating for a balance between technological innovation and ethical accountability, this paper highlights opportunities for sustainable and responsible IoT development that upholds user rights and societal values.

## Review Highlights

• The paper thoroughly explores the multifaceted ethical and legal dilemmas posed by IoT-enabled home environments, emphasizing privacy, security, consent, and regulatory compliance.
• It provides a balanced discussion of current industry practices and government initiatives, alongside proposing best practices and legal frameworks for fostering sustainable IoT growth.
• Real-world examples enrich the paper, illustrating the practical implications of IoT challenges and demonstrating the effectiveness of proposed solutions in addressing critical issues.

## Graphical abstract

**Figure:**
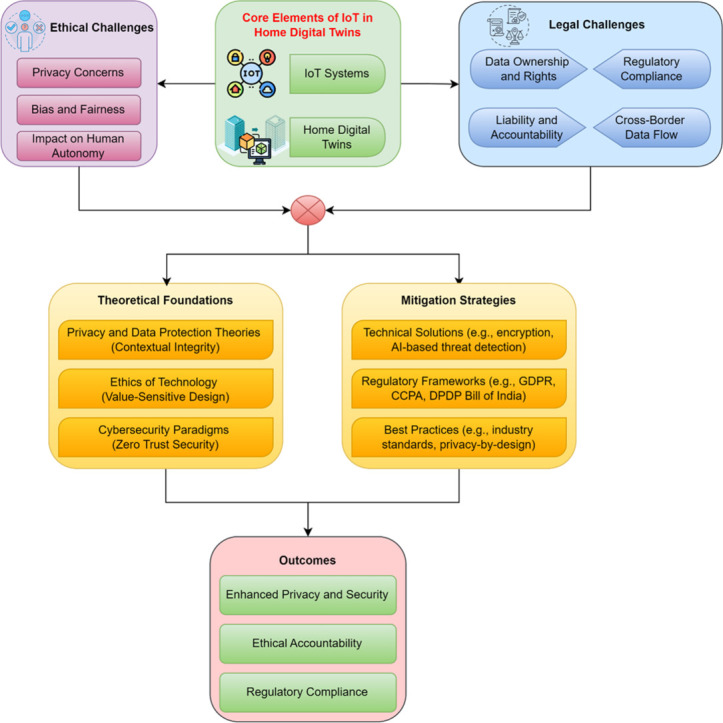

Specifications table

**Table:** 

Subject area:	Engineering
More specific subject area:	*IoT in Home Digital Twins*
Name of your method:	Ethico-Legal IoT Compliance Framework (ELICF)
Name of the reviewed methodology:	*Privacy by Design, Transparency, User-Centric Design, Regular Audits and Updates, Anonymization of Data*
Keywords:	*Internet of Things, Home Digital Twins, Privacy, Data Security, Trust, Anonymization of Data, Cybersecurity.*
Resource availability:	*Not applicable*
Review question:	***Review Questions:*** 1. *What are the primary ethical and legal challenges associated with IoT in home environments, and how do they impact user privacy and security?* 2. *What industry best practices and government regulations are currently addressing these challenges, and how effective are they in ensuring compliance and fostering trust?* 3. *How do the case studies highlighted in the chapter illustrate the real-world implications of ethical and legal issues in IoT applications?* 4. *What are the proposed solutions to mitigate risks like data misuse, unauthorized access, and cyber-attacks in IoT systems?* 5. *How do tracking technologies differ in terms of privacy risks, data anonymity, and vulnerability to misuse and attacks?* 6. *What role do legal frameworks like GDPR, CCPA, and other international laws play in shaping ethical IoT development?* 7. *How can best practices such as data minimization, encryption, and regular audits contribute to sustainable IoT growth while balancing privacy, security, and transparency?* 8. *What are the challenges of cross-border data flow in IoT ecosystems, and how can they be addressed effectively?*

## Background

The rationale for this review lies in the tendencies toward integration of the IoT into the home environment, as well as the augmentation of ethical and legal challenges. Smart homes through IoT can be convenient, and efficient, and there is a need for little interjection as it automates the living spaces. Still, then again, it comes with new issues as what relates to data privacy, security, and ownership over technology products. This review will try to address the existing gap between technology innovation and the protection measures that must be taken to support the sustainable development of IoT systems. Because the IoT has developed so quickly, questions have been raised about its legal requirements, safety standards, and even its ethical design. The subject and its real-life implications, as evidenced by privacy issues and cyber-attacks, cannot be solved he said merely. This review aims to draw practical lessons for developers, policymakers, and end-users from the existing architectures and state-of-the-art research on IoT to build trust and ensure accountability in IoT systems. The setting for this review fits well in with the general trends for the adoption of IoT solutions as part of digital transformation initiatives worldwide, with India and similarly positioned emerging economies as the leaders in IoT implementation. Issues related to the cross-border flow of data, legal mechanisms as well as the ethical aspect highlighted in the paper demonstrate the international and connected nature of IoT environments. Moreover, the use of tables and case instances makes the chapter even more reflective and compares the main problems outlined in the ultimate chapter. Through such concerns, the review expects to help foster positive progress in the IoT while still being responsible for privacy, security, and transparency needs which will in turn be beneficial to both the society and the IoT business*.*

## Method details

### Introduction

Today, with the pace of evolution brought on by technology, the notion of home has been flipped upside down—transformed at its core—due to digital. IoT — The Internet of Things, is one of these solutions that enhance how we interact with the world offering user-friendly environments smartly ready for human experience. Promising IoT application: Home Digital Twins, which combines physical and digital worlds for smart home automation, energy management, and personalized living ecosystem. IoT involves Physical devices that all have sensors built into them, meaning they can collect data, and connect it to the internet for processing (IoT is turning dumb devices smart) This ecosystem comes to an entirely new level when it comes to Home Digital Twins [1]. A Digital Twin is a virtual copy of a physical system, constantly updated with real-time information. Unveiled as housing technology, it generates a real-time digital twin of a home capturing the state of every connected device or system in the infrastructure of a household.

### Brief overview of IoT in home digital twins

The emergence of Home Digital Twins is changing how homes are controlled, monitored, and optimized. IoT provides more control over the living environment than ever before: everything from adjusting lighting and climate control to security systems to predictive maintenance on household equipment. Meanwhile, various smart appliances and devices like thermostats, refrigerators, and security cameras work together as part of a single, connected network in the house that actually knows how to not only follow instructions but can also integrate with its human counterparts, learn from them (in some sense), adapt based on their behavior and predict their needs. It is more sustainable than it could ever be by delivering a smattering of convenience, and that reality extends to the very idea of Home Digital Twins. IoT- enabled twins can optimize energy consumption by analyzing patterns, and adjusting in real-time to save energy costs and environmental impact. For example, smart sensors that can identify vacant rooms and modify heating or cooling as necessary for the space so energy is not wasted. The potential applications extend beyond energy efficiency to healthcare, entertainment, and safety. These wearable health monitoring devices combined with Home Digital Twin will alert the persons about the health anomalies or emergencies. Similarly, real-time surveillance and predictive maintenance create much safer environment by detecting problems such as gas leaks or electrical issues before they actually become dangerous.

However, as the spread of IoT proliferates more depth in our understanding and utilization of how such resources are interacted with — it also clearly raises some critical questions on ethics and legality. With these systems capturing and processing increasing amounts of personal data, questions over privacy, security, and data ownership naturally follow. Although the transition of homes into digital domains is instrumental, it needs to be heavily scrutinized through a lens of ethics and law to provide structure in this new age [2]. The purpose of this paper is to investigate the complex privacy aspects linked with Home Digital Twins on IoT. We hope that this deep dive into data privacy, security risks, and compliance can provide a better perspective of the problem and some potential solutions for the future of smart homes.

#### Importance of addressing ethical and legal challenges

**Fig. 1:**
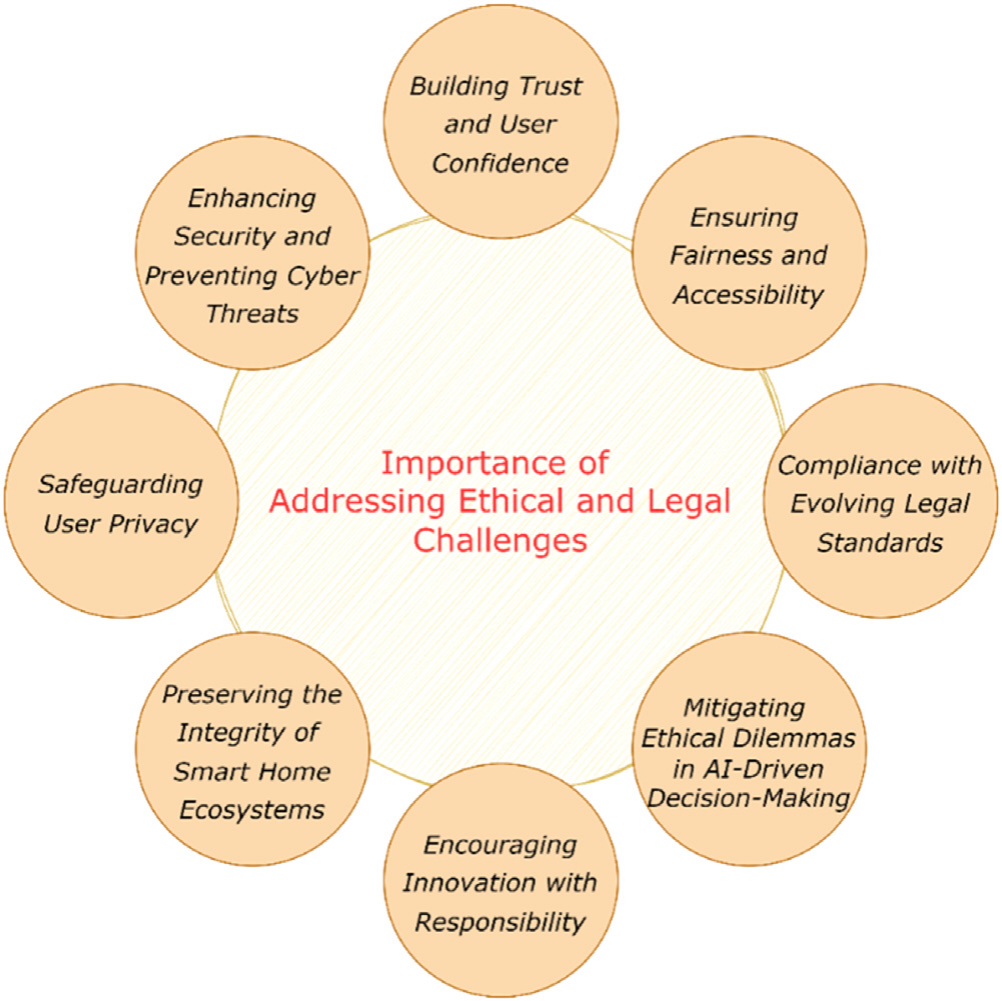
Importance of addressing ethical and legal challenges.

Ethical and legal challenges become ever more urgent as IoT technology becomes an integral part of our everyday lives, where Home Digital Twins are concerned. These components of interconnected devices and systems add a lot of value, but they also introduce security risks that threaten the integrity of smart home ecosystems as shown in Fig. 1. These considerations reinforce the importance of addressing these challenges:

***Safeguarding User Privacy:*** IoT devices in Home Digital Twins generate numerous data and some of them are so sensitive (personal preference, daily activity schedule, health information) that users have to protect themselves from the knowledge of 3rd parties [3]. In the absence of appropriate privacy measures, this data can be accessed and misused by cybercriminals, advertisers, or unauthorized third parties. It is not just about following the law, but an ethical responsibility to protect people from constant surveillance, constant profiling and abuse of personal data.

***Enhancing Security and Preventing Cyber Threats:*** When it comes to securing IoT systems from being hacked or manipulated, it can be quite vulnerable because if any one of the components is compromised, the whole system will probably fail [4]. Malicious activities such as data breaches, ransomware, and device hijacking can jeopardize the safety of Home Digital Twins as well as its functionality. To mitigate these risks, protections against security issues should be addressed at the earliest, with strong legal and ethical frameworks in place to ensure the robustness of smart home systems.

***Building Trust and User Confidence:*** Expose parts of your workings ethically to the end user. One thing is for certain, consumers are not going to touch IoT-enabled Home Digital Twins unless they have assurance that data is treated with care and their security concerns are sustained. Solving these problems solidifies the relationship between technology providers and end users, and sets more aggressive grounds for wide-scale adoption that leads to the long-term sustainability of the tech-dependent world.

***Ensuring Fairness and Accessibility:*** Ethical practices must be fair and inclusivity. The systems of the IoT should be designed in a way so as to ensure equitable service for populations from different demographics thereby preventing an algorithmic or design that may inflict unintentional bias and disproportionately favour one group over the other. The guidelines benefitting from Home Digital Twins could be potentially extended to the populations at large if legal frameworks were employed reinforcing them, in order to promote societal equity and filtration off of digital divides.

***Compliance with Evolving Legal Standards:*** As data protection and consumer rights come to the forefront of privacy law, governments and regulatory bodies around the world are debating even more stringent laws regarding IoT usage and data management. Proactively taking steps to address these legal requirements can mitigate the risk of costly penalties, legal disputes, and reputational damage. In addition, compliance guarantees alignment with world-level norms and standards for the facilitating of IoT technologies across the globe.

***Mitigating Ethical Dilemmas in AI-Driven Decision-Making:*** Most Home Digital Twins rely on AI-driven decision-making systems, which can lead to ethical dilemmas. More fundamentally, who is accountable if an AI system does not stop a security breach or provides wrong predictions? This necessitates a transparent ethical framework governing the use of AI in IoT systems to help respond to these challenges while ensuring accountability and transparency.

***Encouraging Innovation with Responsibility:*** Developers and researchers can innovate responsibly—building in protections from the start by addressing ethical and legal challenges. That's one way to reduce risks as well as promote the technology should move forward in a way that parallels human and legal norms. Instead of just creating cool tech, responsible innovation aims to encourage a sustainable technology ecosystem that serves both individuals and the community at large.

***Preserving the Integrity of Smart Home Ecosystems:*** Home Digital Twins undermine the integrity of Smart Home ecosystems — If failure to address these challenges, it will lead to fragmented regulatory landscapes on a global scale and significant legal disputes and user backlash. Proper deployment of these technologies and other strategies to address ethical and legal challenges safeguard the future of these technologies by ensuring they are sustainable and integration into everyday life.

#### Expected outcomes

The anticipated contributions and impact of this survey is outlined in this section for the ethical and legal challenges concerning IoT in home digital twins. This study focuses on addressing the concerns for privacy, regulatory frameworks, and the user trust to give actionable insights that are beneficial for policy makers, developers, and the users. The defined solutions concerning bridging the gap between technology innovation and moral responsibility for IoT adoption led to the possibility of a sustainable and secure IoT adoption. Some of the expected outcomes of this work are provided below.

1. **Comprehensive Understanding of Ethical and Legal Challenges**: The study attempts to supply an in-depth breakdown of such ethical concerns as the likeliness of user privateness, consent and ownership of information, along with law issues like the necessity of applicable guidelines and the duty for system failures. It identifies the critical vulnerabilities in IoT enabled home digital twins and provides larger journey of ethical and legal landscape around IoT enabled home digital twins and how stakeholders might anticipate and tackle these issues. Such understanding is vital for shaping policies and technologies that will meet social values.
2. **Framework for Ethical IoT Development**: By exploration of best practices and actual case studies, the study offers actionable directions for the development of IoT systems from a moral perspective. Data minimization strategies, user consent mechanisms, and robust security measures are supposed to be considered. This can be a magnifying glass for developers and organizations to create systems ready to prioritize user trust and long-term sustainability, to all the other selfish minds who want to adopt IoT technology.
3. **Regulatory Insights and Recommendations**: Focusing on the Global regulatory frameworks (GDPR, CCPA, India’s DPDP Bill), the findings make a case for harmonizing regulation across sectors. It also provides customized guidance to harmonize these regulations for seamless compliance, thus helping to remove unnecessary operational barriers to global IoT deployments. These insights can foster responsible innovation, as they not only aid policymakers in making informed decisions but also help organizations adjust their practices in accordance with changing legal expectations.
4. **Pathways to Secure IoT Ecosystems**: The study discusses key vulnerabilities that are prevalent in IoT systems, such as DDoS and MITM attacks and the other forms of cyberattacks, that IoT can easily fall victim to. The study further investigates mitigation measures like end-to-end encryption and routine security audits to protect user information and improve system robustness. Discoveries like these pave the way for stronger IoT ecosystems better armed to face changing cyber threats, while also protecting users and maintaining their confidence.
5. **Foundation for Future Research and Development**: The limitations and challenges identified in this study may serve as a valuable foundation for further experimental and theoretical research in privacy-preserving technologies, security protocols, and adaptive regulatory models. The implications of these findings encourage a cross-field of research and collaboration to further develop IoT technologies specifically geared toward home environments. These underpinnings propel innovation that is driven by merchandiser development and ethical responsibility.
6. **Promotion of Sustainable IoT Growth**: The study emphasizes on the necessity of incorporating ethical considerations into the development of technology, to prevent IoT systems from maturing irresponsibly at the cost of users’ rights and benefits. It encourages a sustainable model of IoT development by demanding transparency, accountability, and user-centric designs. By building trust with the user, while addressing societal and environmental concerns, this solution helps pave the way for the broader adoption of IoT technologies in home digital twins.

### Ethical challenges in IoT

As IoT nears ubiquity for functionalities that Home Digital Twins (HDTs) require, there are many ethical issues surrounding privacy. With IoT-enabled systems increasingly becoming a part of our daily lives, they collect abundant data and with it sparks crucial ethical questions on how this gathered information is captured, saved, and used. Here, we explore the primary ethical quandaries concerning privacy in IoT ecosystems.

#### Privacy concerns and data security

HDTs and IoT devices process sensitive personal information, including user habits, interests, and behavioral patterns. Whereas this data allows for advanced features, it comes with the risks of surveillance, unauthorized data access, and misuse [5]. If users feel that they are monitored continuously, they may feel uncomfortable, discouraged, and distrustful. In addition, the absence of strong user consent mechanisms worsens this risk, as most terms of service agreements are complex and obscure, and do not allow the user to be aware of how user data is collected, shared, and used. Addressing these issues necessitates transparency in data practices, strong encryption, and user-centric consent practices that give the control back to users to provide their sharing preferences.

**Ethical Issues in Tracking Technologies:** Tracking technologies like sensors and cameras are a major part of many IoT systems as they help monitor and synchronize data computation in real-time details within HDTs. Yet these technologies pose ethical concerns about privacy intrusions, especially in spaces traditionally considered private. Additionally, the absence of data anonymization techniques and secure data trail security compounds the current challenges with data breaches and unwanted use of the data. To track ethically, data collection must be minimized, anonymization can be effective, and control given to users must be meaningful, balancing utility and user privacy.

**Regulatory Compliance and Legal Frameworks:** IoT revival in HDTs has in turn escalated global regulatory interest Laws like the General Data Protection Regulation (GDPR) in the European Union and California Consumer Privacy Act (CCPA) in the United States set rules around data transparency, user consent, and rights to deletion. Such frameworks require developers to build IoT systems that comply with privacy regulations, thereby supporting consumer rights and better data management practices. These standards are in place to maintain legality and user trust for HDTs.

**Ethical Design Principles for HDTs:** Due to the explosion of digital technology, we have moved from static data collection and monitoring to predictive analytics and action-based insights in a matter of years. But there are also ethical considerations regarding user agency and the fairness of algorithmic decision-making [6]. The real-time data in the IoT systems should also be treated as private and should have real-time mechanisms to secure, process, and anonymize them, while still providing autonomy to the users. Further, designers should be aware of societal standards about privacy and user rights, ensuring that the use of HDTs does not come at the expense of fundamental ethical value.

#### Security vulnerabilities

**Fig. 2:**
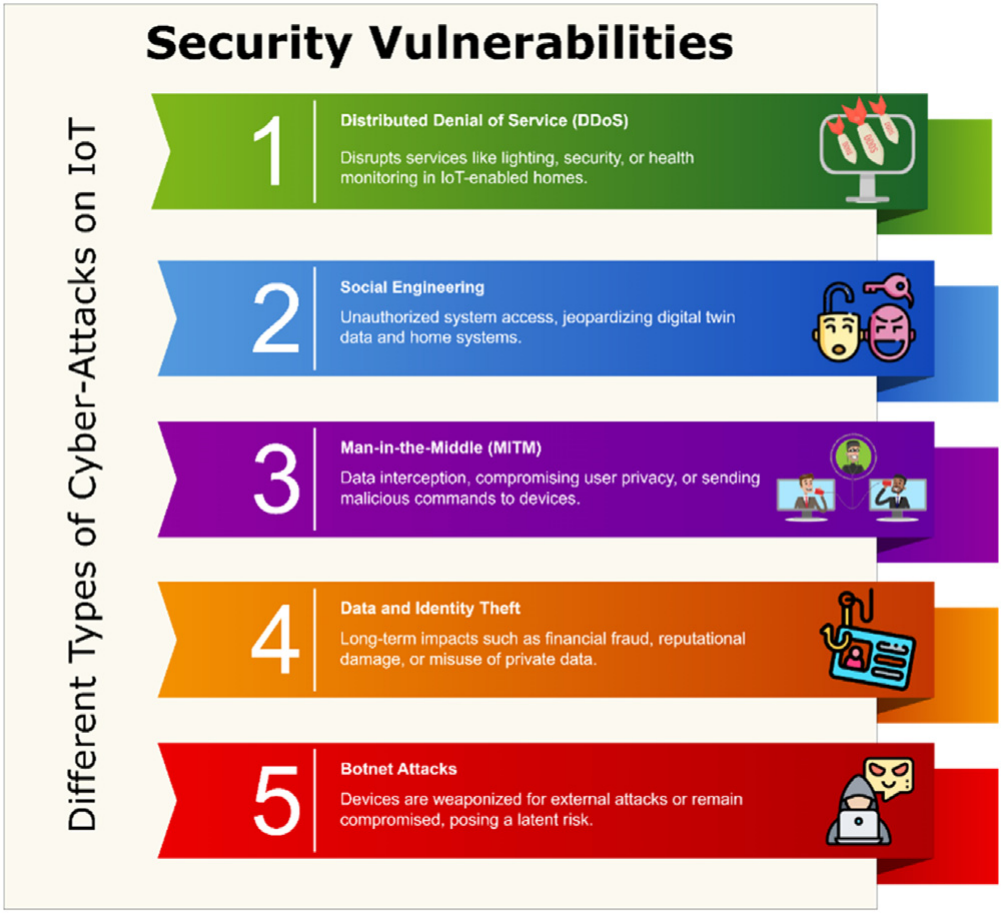
Different types of cyber-attacks on IoT.

IoT has come to play in Home Digital Twins is that these systems are typically at least multiple, if not many more device add-ons per home which makes for a solution set in resiliency and usually de-centralized due to the automated and connected nature of each electrical consumer components [7]. There are so many devices with different types of basic security features, which results in a broad attack surface for cybercriminals as shown in Fig. 2. Knowing the various types of cyber-attacks on IoT systems is essential when they are to be defended adequately.

Table 1 highlights the key cyber-attacks on IoT systems and corresponding mitigation strategies, offering insights into the diverse threats and security measures that can be implemented to safeguard Home Digital Twin environments.

**Table 1:** Overview of key cyber-attacks on IoT and their mitigation strategies.

Cyber-Attack Type	Key Characteristics	Impact on IoT Systems	Mitigation Strategies	Real-World Examples
**Distributed Denial of Service (DDoS)**	Utilizes botnets of compromised IoT devices to flood target systems with traffic, causing service unavailability.	Disrupts services like lighting, security, or health monitoring in IoT-enabled homes.	Traffic filtering, load balancing, firewalls, and frequent firmware updates.	**Mirai Botnet Attack:** Took down major websites like Twitter and Netflix by exploiting IoT devices.
**Social Engineering**	Exploits human vulnerabilities via phishing or fake messages to gain access or trick users into revealing sensitive information.	Unauthorized system access, jeopardizing digital twin data and home systems.	User training, multi-factor	**Shodan Exploitation:** Attackers used phishing to exploit IoT devices found on the Shodan search engine.
**Man-in-the-Middle (MITM)**	Intercepts and sometimes modifies communication between IoT devices, exploiting weak encryption and authentication protocols.	Data interception, compromising user privacy, or sending malicious commands to devices.	Enforcing encrypted communication protocols (e.g., TLS) and implementing strong authentication mechanisms for devices.	**Nest Thermostat Hijack:** Attackers intercepted insecure communications to manipulate smart home devices.
**Data and Identity Theft**	Targets sensitive personal information collected by IoT devices, including health records, behavioral data, and financial details.	Long-term impacts such as financial fraud, reputational damage, or misuse of private data.	Encrypting data at rest and in transit, regular system audits, and using tamper-proof storage methods.	**Target Data Breach:** Hackers accessed customer data through vulnerable IoT-connected HVAC systems.
**Botnet Attacks**	Infects IoT devices with malware, turning them into bots that can perform malicious activities like DDoS attacks or network intrusions.	Devices are weaponized for external attacks or remain compromised, posing a latent risk.	Deploying endpoint security solutions, updating firmware/software regularly, and using intrusion detection systems to monitor for abnormal behavior.	**Bashlite Botnet:** Compromised over 1 million IoT devices to execute large-scale DDoS attacks.

***Different Types of Cyber-Attacks on IoT:***

• *Distributed Denial of Service (DDoS) Attacks:* DDoS attacks take advantage of the interdependence of IoT gadgets to deluge a target framework with traffic from various sources making it inaccessible. These attacks are often launched from botnets of compromised devices, such as poorly secured IoT devices. Home digital twins can be easily disrupted by DDoS attacks, preventing services such as automated lighting, climate control or security systems from operating correctly [8]. Inability to use alarms or health monitors, which can potentially cause harm to physical safety Mitigation Strategies is through traffic filtering and load balancing practices. Using device-level security technologies, such as firewalls and firmware updates.
• *Social Engineering:* Social engineering involves the manipulation of human behavior to compromise a system. These typically take the form of phishing emails or otherwise fraudulent messages that trick you into revealing information, such as login credentials. Social engineering affecting home digital twins including unauthorized access to the digital twin, subsequently jeopardizing user data and home systems. Tailoring device configurations to make them vulnerable or malfunction [9]. Some social engineering countermeasures might include user training in how to recognize phishing and other forms of social engineering. Protect user accounts with multi-factor authentication (MFA).
• *Man in the Middle (MITM) Attacks:* IoT networks are prone to Man-in-the-Middle (MITM) attacks, which intercept and, in some cases, modify communications between devices. It could be through attacks such as listening to sensitive data or malicious commands, jeopardizing the integrity and security of the system at risk. MITM attacks in case of Home Digital Twins could easily reveal private data such as user preferences or surveillance video footage and turn IoT devices into a means of extra-legal control, thus harming either safety or “basic” service provided to customers [10]. In order to prevent such problems, it is of paramount importance to establish consistent protocols for encrypted communication (e.g. using TLS) and means of authentication at both device levels (to protect against collusion and mis-identification) in order to mitigate some risk this will bring with it account termination times — where by new devices simply cannot be easily added without prior whitelisting.
• *Data and Identity Theft:* IoT systems are also constantly collecting a lot of sensitive personal data and are therefore key targets for attackers who can opt to steal this information or the identity behind it. In Home Digital Twins, such attacks can result in compromised user personal data like health records, behavior or financial details with long-term effects like financial fraud or reputational loss [11]. The risk can be minimized by encrypting all data, in rest or while being transported from one point to another such that it is tamper-proof and remains unreadable if intercepted by unauthorized persons, and also carry out regular audits for identifying threats and vulnerabilities in the system.
• *Botnets Attacks:* Botnet attacks, meanwhile, often mean that many of the devices are infected by malware and under control of one or a few attackers — likely to launch other attacks (such as Distributed Denial of Service (DDoS)), or to carry out malicious operations inside the network. In Home Digital Twins, these attacks are highly impactful; devices can get weaponized to attack external targets or other elements of the system. Problematically, these devices could also remain mostly out of operation in a compromised state, leading to a latent security risk that owners may not even be aware is there. The severe threats can be prevented by deploying endpoint security solutions which can detect and handle the malware also firmware’s and software must be updated frequently to respond known vulnerabilities and restore the defense of the system.

#### Bias and fairness

Algorithmic and bias-related concerns, as IoT systems incorporated into Home Digital Twins decide these preference-based and personalized space settings. Additionally, algorithmic biases may result in discriminatory results and an uncritical reliance on these systems could reduce the agency of humans to make choices and carefully consider their decisions.

##### Issues related to algorithmic biases

Algorithm bias occurs when AI systems yield prejudiced or unfair results that are formed typically from design, training data, and operations. These biases can take many forms across any of the myriad IoT systems that resonate within Home Digital Twins. For example, biased training data might cause algorithms to discriminate in favor of specific demographic groups or tastes — which could easily lead to discriminatory user experiences: a home thermostat may set different energy usage profiles for households with the same practice because the underlying data was biased. Exclusionary practices are also a problem since biased facial recognition and voice command systems can alienate users based on race, gender or, accent, leading to limited access to IoT capabilities. Moreover, IoT learning feedback loops based on user actions can reinforce and amplify the natural biases, producing auto-replicating loops that further disadvantage those users [12]. Strategies include diversity among the data scientists creating these systems, performing a variety of bias audits and impact assessments periodically, enforcing fairness metrics during algorithm design to limit differential outcomes, and supporting the ground truth.

#### Impact on human autonomy

Upon introducing IoT and automation into Home Digital Twins, a contradictory situation is brought about in which the two forces work hand-in-hand to not only make existence extremely convenient and efficient but also to compromise human agency. This over-reliance on automation raises a host of ethical issues. The major problem, of course, is the erosion of critical thinking when users rely upon automated decisions without questioning whether those decisions are right — with the inevitable result that they are no longer able to determine if a situation requires critical thought. For instance, an intelligent security system may decide to allow or deny access on the basis of its algorithmic discretion which cannot be overridden by users even when it makes a wrong call. Loss of control is another worry — having smart IoT systems take actions without enough user input, changing or crashing our things may mangle user preferences, leading to a blunt experience and less ownership over their living environment. On the other hand, users might become helpless in case of system breakdowns or malfunctions if they solely depend on IoT systems and do not know how to do some of the tasks manually. Systems must be designed that prioritize user participation and model behavior in a transparent manner to reduce these risks [13]. Allowing for manual overrides or implementing human-in-the-loop mechanisms can preserve user control; promoting digital literacy makes sure that a user is able to comprehend and critically interact with IoT systems.

##### Over-dependence on automation and decision-making

Seamless integration of IoT devices into Home Digital Twins is a potential risk in terms of over-dependence on automation and decision-making if not carefully implemented. With increasingly of users trusting their automation systems with both important and mundane decisions, the upside that comes from optimization may include some downsides. Reliability risks are a main problem of dependence: if people depend increasingly on IoT systems, household operations are at increased risk of disruption in the event that devices fail, get hacked, or outdated [14]. In those same instances, there would be significant ethical dilemmas due to AI-driven actions, particularly when automatic decisions have significant ethical inferences (for example choosing who receives attention first during an emergency). In these cases, the reliance on IoT systems might as well transfer guilt from humans to machines. Worse, long-term dependence on automation will likely erode those essential human skills – like knowing how to balance the home's power use or diagnose a security problem – because if the tech does it all, why should we bother learning? While falling back to usable systems in the absence of IoT functionality is vital, user participation through bespoke settings and alerts for when their action is required and maintaining regular updates on systems will increase reliability while reducing the risk associated with technological obsolescence.

### Conceptual framework

**Fig. 3:**
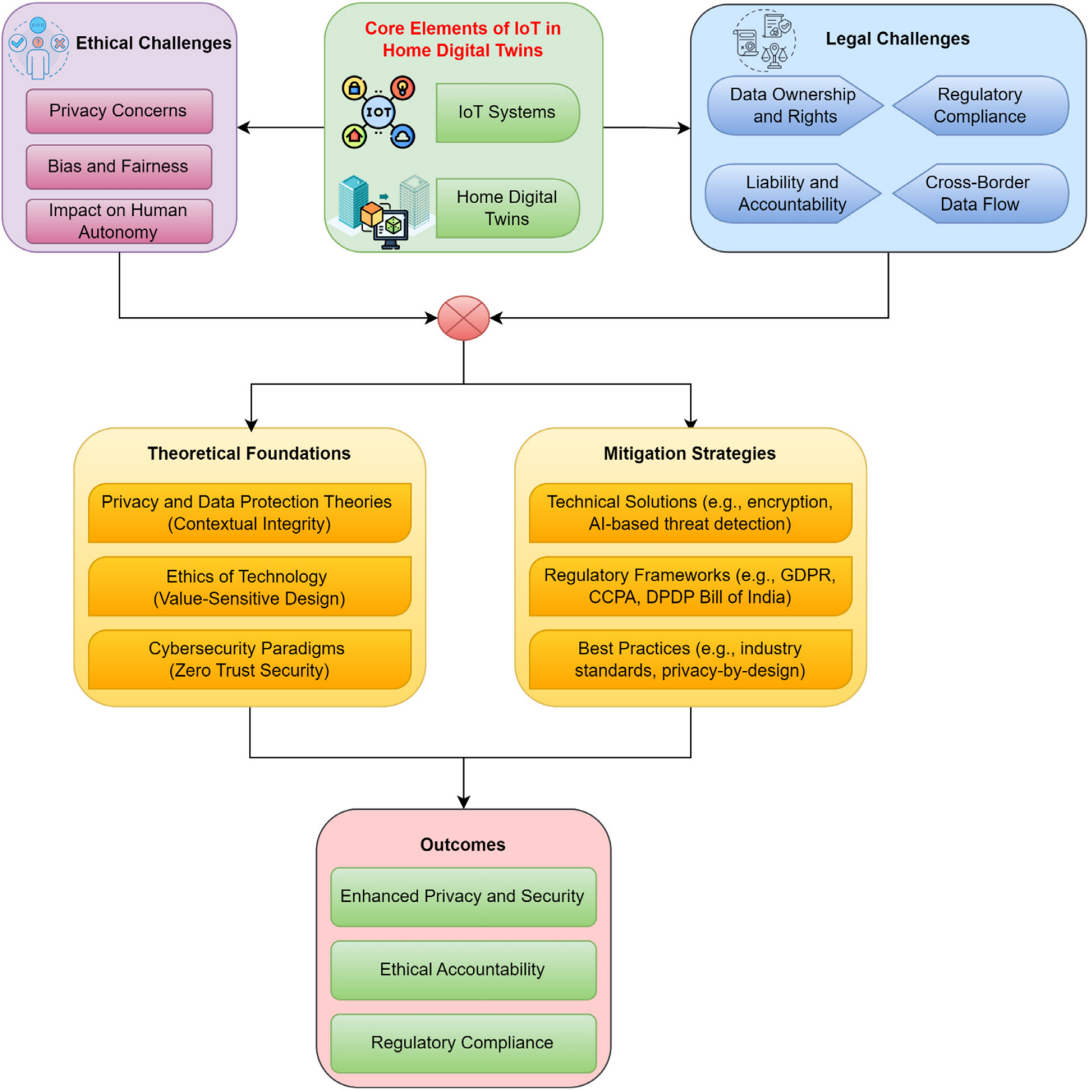
Conceptual framework for addressing ethical and legal challenges in IoT-enabled home digital twins.

The integration of Internet of Things (IoT) technologies into Home Digital Twins (HDTs) marks a transformative step in creating intelligent, responsive, and interconnected home environments. These technologies enable real-time data exchange and control, while HDTs serve as dynamic, virtual representations of physical home environments. Together, they form the foundation of intelligent home ecosystems, providing automation, energy efficiency, and enhanced user experiences. However, this advancement also introduces significant ethical and legal challenges, requiring a structured framework to address these issues effectively. This conceptual framework outlines the core components, theoretical foundations, and interrelationships necessary to understand and tackle these challenges, culminating in actionable outcomes as shown in Fig. 3. At the core of the framework lies the intersection of IoT systems and Home Digital Twins. The dynamic interaction between these elements facilitates seamless integration and innovation within smart home environments. However, the massive data collection capabilities of IoT systems give rise to ethical concerns. Privacy intrusions and unauthorized data usage represent critical challenges, while algorithmic biases embedded in IoT-driven HDTs may lead to unequal treatment or exclusion of specific user groups. Additionally, over-reliance on automated systems can erode human autonomy, creating dilemmas in decision-making and control. These issues underline the necessity of addressing ethical concerns to maintain user trust and inclusivity in smart home systems.

Legal challenges further compound the complexity of IoT adoption in Home Digital Twins. The ownership and control of data generated within these ecosystems remain contentious, particularly when considering cross-border data flows and regional regulatory requirements. Compliance with frameworks such as the General Data Protection Regulation (GDPR), the California Consumer Privacy Act (CCPA), and India’s Digital Personal Data Protection (DPDP) Bill is crucial but often challenging due to their variability and evolving nature. Questions of liability and accountability for system failures or data breaches add another layer of complexity, making it essential to establish robust legal safeguards that adapt to diverse operational contexts. The framework is grounded in well-established theoretical foundations. Ethical technology principles, such as value-sensitive design, advocate for embedding ethical considerations directly into the design and development processes of IoT systems. Privacy and data protection theories, including contextual integrity, emphasize aligning information flows with user expectations and societal norms to preserve trust and compliance. Cybersecurity paradigms, such as zero-trust security, stress the importance of continuous verification and robust encryption to protect sensitive data and maintain system integrity. These theories provide a comprehensive basis for understanding and addressing the challenges inherent in IoT-enabled HDTs.

To mitigate these challenges, the framework proposes a multifaceted approach that combines technical solutions, regulatory frameworks, and best practices. Advanced encryption techniques, AI-driven threat detection systems, and zero-trust architectures are critical for securing sensitive data and preventing unauthorized access. Harmonizing global standards, such as GDPR, ISO/IEC, and ETSI EN 303 645, ensures consistent compliance and fosters user trust across jurisdictions. Best practices like privacy-by-design, routine security audits, and user-centric customization further enhance system resilience and ethical accountability. By integrating these strategies, the framework ensures a proactive and adaptive response to emerging challenges in IoT ecosystems. The interrelationships between these components are central to the framework's effectiveness. Ethical challenges are directly connected to technical solutions and theoretical insights to ensure responsible innovation. Legal challenges, on the other hand, necessitate collaboration between regulatory frameworks and best practices to streamline compliance. These elements work together to achieve the overarching outcomes of the framework, including enhanced privacy and security, ethical accountability, and simplified regulatory compliance. The ultimate goal of this conceptual framework is to foster a secure, ethical, and legally compliant IoT ecosystem within Home Digital Twins. By addressing the challenges outlined, the framework aims to protect user rights, build trust, and enable sustainable innovation. The integration of ethical principles into technological advancement ensures that Home Digital Twins become transformative tools that align with societal values while safeguarding individual freedoms. This structured approach paves the way for a future where IoT-enabled HDTs achieve their full potential without compromising fundamental ethical and legal standards.

### Legal challenges in IoT

**Fig. 4:**
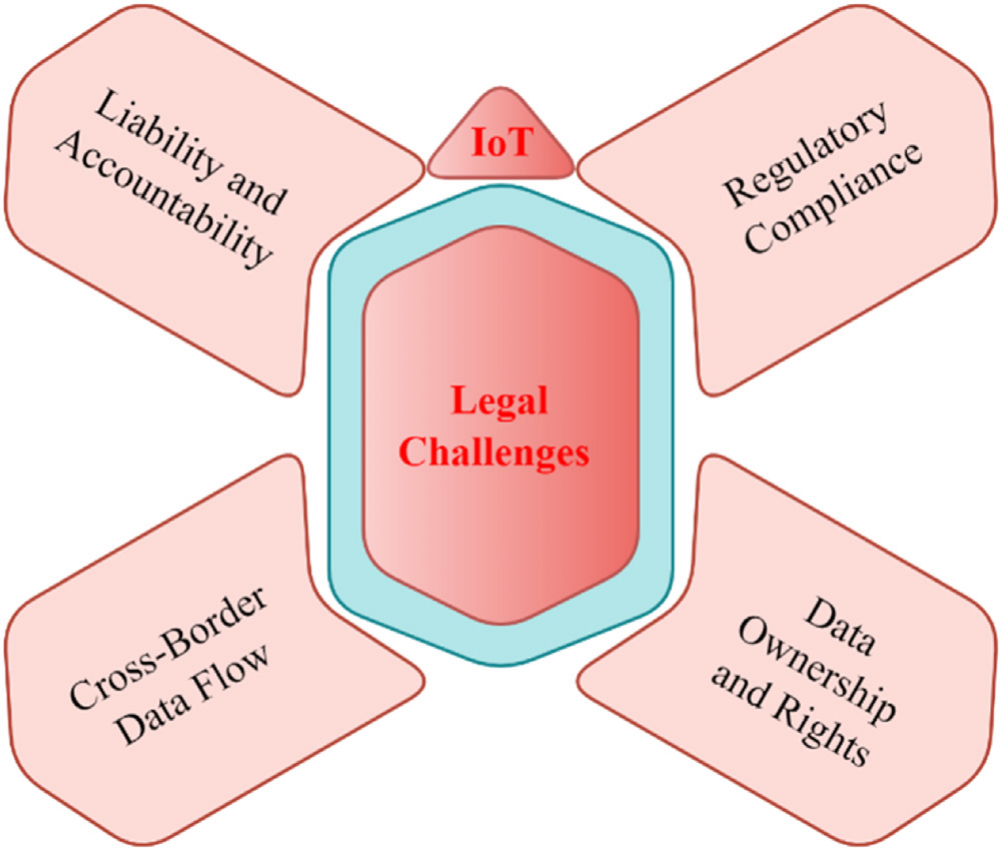
Legal challenges in IoT.

Legal challenges in IoT are multifaceted and evolve rapidly alongside the growth of connected devices and smart technologies. With the different ways IoT systems are integrated with personal, commercial, and industrial spaces, thoughtful legal problems appear as to that possesses data, along with privacy issues influencing everything from software security to compliance-oriented developments as shown in Fig. 4. One of the key debates continues to be around ownership, sharing and/or protection of generated data acquired by IoT devices, with the end user typically lack control and transparency dimensions over their data. Furthermore, the legal frameworks are less straightforward given the encumbered liability for damages triggered by malfunctioning devices or security breaches — particularly when device manufacturers and service providers are involved. As everyday life is increasingly shaped by IoT technologies, the law needs to catch up and handle the resulting challenges effectively, balancing innovation against user rights and public safety on the side of caution.

#### Data ownership and rights

One of the most important legal challenges facing IoT is the ownership and rights of data. Home Digital Twins has lots of data from IoT devices, including personal, behavioral, and environmental information. The question of who owns data like this — does it belong to the person generating it, the manufacturer of the smart device, or third parties involved in processing that data? — has huge legal implications. All of this means that users may not even know how their data will be gathered, used or, shared, leading to a whole new set of problems around data control and use-rights [15]. However, the speed of technological development is often unfathomable for legal frameworks, which historically have been established as a response to specific challenges driven by economic or political necessity and therefore are not always fit for new concerns around consent, access, and particularly data portability. In addition, there is a legal question of whether data generated by IoT devices should be classified as personal data or more generally under some other category.

##### Devices to enhance user experience

IoT devices are created to make the lives of people connected with them easy and convenient due to automation, personalization, and customization but at the same time, they raise a lot of legal issues. This includes matters of user consent, product liability, and regulatory approval. Manufacturers are legally obliged to ensure that the devices are secure, respect user privacy, and comply with all relevant laws. That responsibility to keep the device in a safe and secure manner and protect the data flows down to legal discussions when a device is compromised or an individual user suffers harm. The inclusion of IoT devices in consumers' daily lives also calls into question consumer autonomy, control, and willingness to accept the specifications required for an improved experience. The law is famously slow to catch up with technology, and legal frameworks around liability, consent, and user rights.

#### Regulatory compliance

For IoT, regulatory compliance refers to those mechanisms that protect user privacy in data collection, storage, and processing while respecting fundamental human rights. Global standards for data protection have been established with regulations like the General Data Protection Regulation (GDPR) in Europe and the California Consumer Privacy Act (CCPA) in the United States. Both require user consent, transparency in data collection/handling, and the right to have or remove personal data (strictly under GDPR and with applicable limits in the CCPA). Some countries such as Canada, India, and Australia have passed privacy laws. These laws make it impossible for IoT developers and service providers to design solutions that allow them to neglect privacy and security. This kind of compliance not only prevent potential legal consequences but also fosters overall trust with users for practicing responsible data management.

Table 2 compares five major regulatory frameworks: The survey involved five laws, including GDPR, CCPA, DPDP, Australia’s Privacy Act, and Singapore’s PDPA, and was evaluated based on data-on-data privacy, user consent, and cross-border data flow. CCPA can be considered the least suitable, as it does not address all three challenges, while the GDPR covers all of them. In the case of India, the DPDP has poor coverage of user consent and restricted cross-border data flow. As good as Australia’s Privacy Act 1988 with principles of privacy and consent is, it does not fully tackle cross-border concerns This calls for more harmonization of IoT regulations on a global scale.

**Table 2:** Comparison of regulatory frameworks for IoT privacy, consent, and cross-border data flow.

Regulation	Data Privacy	User Consent	Cross-Border Data Flow
General Data Protection Regulation (GDPR)	✓	✓	✓
California Consumer Privacy Act (CCPA)	✓	✓	✗
Indian Data Protection Bill (DPDP)	✓	✗	✗
Australia's Privacy Act	✓	✓	✓
Singapore Personal Data Protection Act	✓	✓	✗

✓ = Effectively Addresses the Challenge, ✗ = Limited or No Coverage

The Table 3 on the "Pros and Cons of Key Regulatory Frameworks" highlights GDPR's comprehensive data protection approach, CCPA's consumer empowerment focus, DPDP's data localization emphasis, Australia’s balanced yet outdated Privacy Act, and Singapore’s PDPA's user-centric model, illustrating the strengths and limitations of each framework in the context of IoT privacy and data management.

**Table 3:** Pros and cons of key regulatory frameworks.

Regulation	Pros	Cons
General Data Protection Regulation (GDPR)	- Comprehensive and stringent framework for data privacy. - Mandates user consent, transparency, and "right to be forgotten." - Supports cross-border data flows with safeguards.	- High compliance costs, especially for SMEs. - Ambiguities for non-EU countries interacting with EU residents. - Severe penalties for non-compliance.
California Consumer Privacy Act (CCPA)	- Empowers consumers with rights to know and request deletion of data. - Flexible compliance structure for U.S.-based IoT companies.	- Limited scope, applies only to businesses meeting specific criteria. - Lacks provisions for cross-border data transfers and strict data minimization.
Indian Digital Personal Data Protection Bill (DPDP)	- Emphasizes data minimization and purpose limitation. - Promotes data localization and sovereignty.	- Weak user consent mechanisms with reliance on implied consent. - Restrictive cross-border data policies limit global IoT integration.
Australia’s Privacy Act	- Balances user consent, data minimization, and cross-border provisions. - Encourages transparency and accountability.	- Requires modernization to address IoT and real-time data challenges. - Limited penalties reduce enforcement strength.
Singapore’s Personal Data Protection Act (PDPA)	- Strong focus on user consent and accountability. - Simplified compliance structure facilitates regional IoT adoption.	- Lacks robust cross-border data transfer provisions. - Challenges in addressing high-volume, real-time IoT data.

##### International context and harmonization

One of the most significant challenges presented by the fragmentation of regulations in regions is the global IoT ecosystem. Lack of harmonization in data policies at a global scale, such as the strict cross-border data transfer rules laid out in GDPR, but not covered by CCPA, makes IoT solutions difficult to implement seamlessly in all regions. Regional regulations, such as India’s DPDP, with its unique data localization focus, restrict global scalability even further, while frameworks such as Australia’s Privacy Act and Singapore's PDPA, need to evolve to keep pace with the rapid pace of change in the IoT space. International harmonization of data protection laws is needed to overcome these issues. Unified standards that strike the balance of privacy, security, and innovation can simplify compliance, lower costs, and facilitate international collaboration. With adherence to these budding standards by the IoT developers, IoT solutions can easily be developed keeping in mind the user rights along with encouraging innovation and scalability on the worldwide market.

#### Liability and accountability

As IoT systems are formed by an unprecedented interconnection of devices and a multiplicity of stakeholders (manufacturers, service providers as well as end users), discussing liability and accountability is not only complex but also integrative. Because of the complexity associated with contributing parties and roles in designing, deploying, and operating IoT systems responding to a system failure can prove difficult [16]. For instance, if a device fails and causes property damage or a breach in security the device manufacturer can be held accountable for design failures; while the service provider or cloud platform hosting data can be liable for cybersecurity vulnerabilities. Users seem to bear some responsibility, especially if they do not update software or follow basic security protocols. Legal frameworks are needed to add context to explicit rules for liability of exposure to ensure that users and responsible innovation in the IoT space are protected by ensuring accountability from all parties.

#### Cross-border data flow

Cross-border data flow is among the significant issues in international IoT deployments because of conflicting data protection laws. However, with IoT devices gathering and storing enormous amounts of personal data, this raises legal questions about what is allowable here as well [17]. The discrepancy between jurisdictions in the standards that they require for data privacy, security, and storage is undeniable. For example, the General Data Protection Regulation (GDPR) of the European Union, imposes strict data transfer rules outside the European Union to require those countries to comply with GDPR standards, while some other countries may have weaker or less mature data protection laws. This poses problems for multinational IoT suppliers, which have to comply with several legal frameworks when handling user data. In addition, data across borders can be made more difficult not only by geopolitical tensions and trade restrictions [18]. Meeting these challenges requires IoT companies to remain compliant with rapidly evolving clear data governance policies and enforce effective security measures so that user data is protected without affecting the seamless operational flow of international IoT.

Table 4 evaluates and compares the effects of multiple legal issues with IoT settings regarding data protection, security, and compliance. This is because DRs have positive effects on both data privacy and security since they compel organizations to store data and process data within the country. However, these requirements raise compliance issues because of these different standards around the world [19]. There are differences in laws and standards for data privacy but they do not necessarily alter the security or compliance value greatly. Manipulations of data transfer restrictions are enemies of data privacy yet they contribute to security by reduction of the mobility of the sensitive data across systems while increasing the level of difficulty in compliance. Encryption standards are good for privacy and security but make the compliance process difficult, especially due to the differences in standards by the region of the world. Jurisdictional issues do not affect data privacy and security directly but give rise to an immense number of compliance issues since they make international data exchange and obtaining legal redress difficult.

**Table 4:** Impact of IoT legal challenges on data privacy, security, and compliance.

Issue	Impact on Data Privacy	Impact on Security	Compliance Complexity
Data Residency Requirements	✓	✓	✗
Divergent Legal Frameworks	✓	✗	✗
Data Transfer Restrictions	✗	✓	✗
Encryption Standards	✓	✓	✗
Jurisdictional Disputes	✗	✗	✗

✓ = Positive Impact (Supports Better Management), ✗ = Negative Impact (Creates Challenges)

### Tracking technologies and related threats

This ability to gather and send data more or less continuously for real-time synchronization is essential to Home Digital Twins, powered by tracking technologies in IoT systems. They monitor and track the behavior, preferences, and total interaction of users with their surroundings by using different devices like Motion detectors, GPS sensors, RFIDs, etc. Although such platforms provide major convenience and personalization advantages, they also come with privacy, data security, and potential abuse issues. The continued existence of these tracking technologies leaves behind a never-ending data spark that can be fanned if the information in which they forcibly inquire is ever accessed by anyone with an inappropriate interest [20]. The rise of IoT attacks and the level of sophistication in the cyber-attacks that are executed on them based on human lifestyle aspects, such as homes, wearable devices (home space), or vehicle type raise much higher risks for using IoT.

The problem is even more acute when you consider the increasing interconnectivity of IoT devices as some tracking mechanisms that operate perfectly well in isolation start to look an awful lot like surveillance when deployed on a network. In addition, if you do not provide effective techniques of anonymization, individuals can be re-identified and become exposed to potential identity theft or profiling. The ethical issues that surround tracking technologies can be termed as a delicate balance of innovation and privacy; the only way out is decentralization/peer-to-peer technology development involving the creation of open systems that put more power into the hands of their users. Table 5 is an attempt to compare several IoT tracking technologies in terms of privacy threats, data randomness, likelihood of data abuse, and susceptibility to cyber assault. GPS tracking has been proven to be very effective in location tracking, however, there is a major issue of privacy invasion since tracking numbers do not need to be anonymous, and it is easily vulnerable to misuse and hacking. Compared to the RFID technology it provides lower attacks and privacy risks but data anonymity is not fully preserved and can be misused [21]. Bluetooth beacons enhance data anonymization but are insecure and can be misused in some situations. Wireless connection tracking is also extremely susceptible to privacy invasion and abuse and constitutes rather low safety against cybercriminal activity. Biometric sensors, while helpful for identification have high concerns of privacy; nonetheless, they have improved data anonymization than other technologies. These tracking methods show that there is a difference in the performance and the degree of threats present in the IoT systems and therefore one has to look at the issue of tracking with regard to privacy and security issues.

**Table 5:** Privacy and security risks of IoT tracking technologies.

Tracking Technology	Privacy Risk	Data Anonymity	Threat of Data Misuse	Vulnerability to Attacks
GPS Tracking	✓	✗	✓	✓
RFID	✓	✓	✗	✗
Bluetooth Beacons	✗	✓	✓	✗
Wi-Fi Location Tracking	✓	✗	✓	✓
Biometric Sensors	✗	✓	✗	✓

✓ = High Risk/Effectiveness, ✗ = Low Risk/Effectiveness

#### Three main types of attacks on tracking devices

***a. Unauthorized Access to Tracking Devices***

Unauthorized access to tracking devices is a significant security risk within the space of IoT since attackers are able to control devices that were made specifically for monitoring and collecting sensitive data. These attacks can occur in a number of ways, such as by exploiting vulnerabilities in the firmware of devices; obtaining weak or stolen credentials, or bypassing security procedures. With unauthorized access, attackers can disable the tracking device, listen to users performing activities, or modify the data being sent which could lead to majority privacy violations. When it comes to Home Digital Twins, this is an alarming scenario considering that with a single breach, attackers can get fed data such as their routines, health data feed, preferences, and behavioral patterns. Information of that kind if not handled carefully could lead to identity theft, fraud, or even worse physical invasion such as accessing a user's household or vehicle without authorization.

One of the reasons unauthorized accesses remains a problem is that IoT devices, most of them without strong security measures, are present everywhere. With how manufacturers and devices vary in terms of security protocols, it also makes ensuring consistent protection that much harder. In addition, users frequently fail to update their device software or modify default security settings, making them susceptible to attack. Occasionally, attackers can use devices with a password (or no password) to eavesdrop on data during transmission and if the encryption method is weak or not in place at all they can recover valuable information [22]. The unauthorized access of tracking devices also compromises the privacy and trust of the Home Digital Twin system. Devices that monitor and control critical functions, such as smart thermostats, alarms, security cameras, etc., could be manipulated to create chaos in a household operation. This could happen if attackers took control of security systems, changed environmental controls, or issued malicious commands to other connected devices leading to the system not operating as it was meant to and potentially falling into a malicious state. This underscores the importance of comprehensive privacy and security safeguards to prevent unauthorized access which would only become more pervasive as IoT devices become increasingly entrenched in everyday life.

Mitigating unauthorized access to tracking devices, therefore, requires a multi-layered security approach [23]. These features enable robust authentication protocols with multi-factor authentication and biometric identity checks to ensure that only authorized users can access the function. By encrypting data in rest and in transit so that if data is intercepted it will be unreadable to attackers. IoT devices should receive consistent updates of patches, to fix any known vulnerabilities and avoid successful exploitation. Users should also be educated on the need to protect their devices in a similar manner, by using strong passwords, switching off any services they do not use and monitoring device behavior for unusual activity. Manufacturers also bear a large responsibility to begin implementing security features in the devices and focusing on secure firmware development as well as using commonly accepted encryption methods. Manufacturers and consumers alike can lessen the risk of illegal access to tracking devices and safeguard the confidentiality and integrity of Home Digital Twins by implementing a proactive security approach.

***b. Attacking the Tracking Device Availability***

The presence of tracking devices is vital for the Home Digital Twins to function properly and provide continuous operation. Such devices provide tracking, collecting, and transmitting data round the clock for real-time decision making and automation required. Nevertheless, these machines could be taken offline by an attack causing it to be non-operational or disrupting its core capabilities. These attacks, known as Denial of Service (DoS) or Denial of Device (DoD) attacks, are aimed at the underlying operating capability of IoT devices that make real-time data transmission impossible, shutting down the whole system. These assaults tend to render devices incapable of serving their intended purpose, either by inundating the device with excessive requests, disabling communication channels, or leveraging software vulnerabilities allowing attackers to tamper with normal device operations. Distributed Denial of Service (DDoS) attacks are one of the main methods available for targeting tracking device availability. This type of attack simply floods an IoT device with more traffic, then it can handle, from a network of compromised devices (a botnet), preventing the device from responding to legitimate requests. As a result, the device can stop responding or go off-line to be extremely slow at sending critical data to other components of Home Digital Twin [24]. An IoT-enabled smart security camera being victim to such an attack might be unable to deliver live surveillance feeds or raise alerts upon detecting unauthorized entries, as a case in point. Similarly, in a smart home ecosystem, where tracking user movement or environmental conditions may be disrupted leading automated processes or potentially bringing your whole system failure.

Attacking tracking device availability is another method of service disruption using the communication protocols or software running on the device [25]. Attackers may attempt to block, disrupt, or modify these signals if an IoT device is using weak or outdated communication protocols. A simple example would be if an attacker targeted Bluetooth or Wi-Fi communication, they could break the connection between a tracking device and the central system, which disrupts services temporarily but is not too damaging in this context. If the devices rely on cloud-based servers or remote services to store data and process, the attacker can target these cloud platforms to isolate those devices by not allowing them to retrieve or transmit any data. These interruptions can prevent the Home Digital Twin system from up-to-date and accurate depictions of the physical environment, countering the efficiency and safety that IoT systems are designed to deliver. Targeting the availability of tracking devices can impact the entire Home Digital Twin solution. A run on deliverable data due to an availability attack breaks the basic promise made by IoT- which will automate, personalize and be able to make intelligent decisions based on predictive analysis [26]. If, for example, environmental monitoring tracking devices go offline because of extreme weather conditions than the entire system's ability to adjust temperature settings or notify users of potential dangerous severe whether events may be impaired which could arguably lead to discomfort or even potentially property damage. For example, loss of a tracking devices which are used to monitor for security inside the home can expose the house open to be burgled. The intelligence could be crippled without this critical data from these machines, translating into lesser user satisfaction and a potential safety risk.

Given the types of attacks possible against tracking devices, a solid security strategy is necessary to prevent them from influencing the availability. The most important is to build resilience mechanisms into the IoT devices so they can continue to work as intended even when attacked. Like, devices can have fallback systems that enable them to use backup servers or redundant systems whenever a failure occurs in the system. In addition, the use of traffic filtering and rate-limiting can prevent DDoS by blocking malicious traffic before it reaches the target device [27]. This also means ensuring that devices are designed with secure communication protocols using encryption and authentication to decrease the risk of unauthorized access and verification of message transfer consistency as well. Both can be largely prevented if proper monitoring and testing of IoT devices are conducted to identify possible security risks and vulnerabilities, which could be taken advantage of by malicious actors. Conduct periodic security audits and firmware updates to close any vulnerabilities, strengthening the resilience of devices. In addition, creating network segmentation enables to isolate vital tracking devices away from the rest of the system and ensures an attack does not trickle on other devices. These initiatives will hopefully help manufacturers and users of tracking devices to better guarantee availability within Home Digital Twins, ensuring seamless operation of IoT systems.

***c. False Data Injection on Tracking***

False data injection attacks over the tracking devices in IoT systems are real threats and they can compromise the integrity and trustworthiness of the entire Home Digital Twin ecosystem [28]. In such attacks, attackers intentionally enter false or deceptive data into the system to contaminate the tracked and transmitted data of IoT devices. We don't necessarily want to disable the devices, but rather change the data they see or communicate to alter the way a system makes decisions or courses of action in an adversarial context. The impacts of false data injection attacks can range from nuanced interferences to complete system collapses, depending on the function of the inaccurate data and how critical decisions are based on it. A common practice for false data injection is to intercept and manipulate the signals between IoT devices and the central control system or cloud platform. One example might be an IoT device in a smart home that records environmental monitoring information such as temperature, humidity, and energy usage; an attacker could then send false data to the good system thereby detecting incorrect normal ranges for the environmental conditions [29]. For example, a thermostat may be fooled into keeping a desired temperature at gangbuster levels when no one is home, but in fact the home itself has heat coming from other rooms or floors. In the worst case, these could result in catastrophic failures such as home systems stopping working, or even worse can be safety hazards; like a leak into the fire alarm system from injected data leading to false alarms.

False data injection like this in security systems will also impact the Home Digital Twin. The cameras and the motion detectors are used to keep an eye on the safety of a house through IoT. However, an attacker could change the data that these devices present, thereby causing the system to misinterpret everyday normal behavior as a potential threat or, conversely, not be able to find suspicious behavior. Under this scenario, either a homeowner can be unnecessarily alarmed or a real intrusion could take place but go unnoticed — the risk could be higher for theft/vandalism. The modification of such data through false injections in security systems can remove most of the trust we put into fully automated IoT applications, with possibly catastrophic consequences for user's safety. Such false data injection attacks can also operate against essential systems which use real-time data to evolve their dynamic decisions [30]. For example, in a smart healthcare application, IoT sensors keep an eye on vital signs such as heart rate, blood pressure, glucose level, etc. If an adversary injects false data in one of the databases without performing a maneuver and misdiagnoses the user it could lead to a serious risk to the user. The consequences of a false data injection attack in this latter case however are not the same inconvenience or security breach, but has direct risk to life or wellbeing of one depending on that system for monitoring and care.

Protecting your IoT systems from malicious injected data will require a multi-tiered approach to both securing the data and verifying the system as a whole. Data validation and verification techniques are one of the key mitigation strategies. Critical data should be checked at multiple observation points before acting upon it to ensure that it conforms to expected norms and patterns. For example, anomaly detection algorithms highlight any anomalies in data behavior that would imply tampering. This is particularly powerful when it comes to large-scale IoT systems that do not get as much human oversight, but for which automated decision-making processes are very dependent on the integrity of the data collected [31]. Encryption and authentication protocols are also very important to prevent false data insertion. This approach ensures that the communication between IoT devices and central control systems is encrypted, which means attackers cannot eavesdrop on or tamper with data in transit. To also use secure authentication mechanisms is such that only those devices that we trust will be able to push their data in, making it less prone for an attacker to introduce fake data from a compromised device. Digital signatures are also a form of cryptographic technique to confirm the data and make sure that its source is trustable.

Redundancy and cross-checking by multiple devices are other important defenses against false data injection. This system can detect variances between different sensors or devices that are monitoring the same data and identify outliers as potential fabricated data. For instance, if two or three smart thermostats in a building report an out-of-the-way temperature difference, it can alert to come for verification (by sensing humans) that this data may be true/false. Particularly in domains like security and health monitoring systems where data can be tampered with, and thus the presence of equivalent data from several diverse sensors can help detect these spurious attempts to inject false data [32]. Finally, there is a way to use machine learning models to identify these staggered patterns of false data injection. These models can enable the learning of normal behavior and promptly pinpoint distinct (irregular) data values that deviate from the predicted pattern. Historical device data can improve the models further, giving broad insight into typical device behavior and uncovering trends that might even be data manipulation.

### Case studies

Here are five real-time examples of ethical and legal challenges in IoT-enabled homes, highlighting different issues such as privacy concerns, data security, and compliance with regulations:

#### Case study 1: artificial intelligence-driven home digital twin

A home digital twin (HDT) was created using artificial intelligence, sensor technology, and virtual reality in this case study [33]. The HDT is designed to be a modern home with offices that cover multiple levels of capabilities. The standalone digital twin, which is then rendered to a detailed 3D model suitable for both visualization and engineering simulation, scalability has been tackled via photogrammetry and polygon optimization. When combined with sensor readings and other external information, such as satellite data, it becomes a descriptive digital twin. However, instead of directly learning the relationship between input variables to the output, DT uses basic statistical tools (like PCA) to evaluate anomalies and provides predictive capabilities, using both data and physics-based modeling. By collaborative filtering learning from user behavior and external datasets, prescriptive DT insights are generated. Potentials for full-scale implementation have been demonstrated with limited autonomous functionalities, such as smart lighting control, and recommendations for such implementation include occupant psychology and behavior. The extensibility of DTs, and their use as a way to advance HDT applications to remote monitoring, simulation, or control, are emphasized through this modular framework.

A Home Digital Twin (HDT) powered by artificial intelligence, sensors, and virtual reality leads to a great ethical and legal challenge. Privacy is among the biggest concerns with combining real-time sensors, satellite imagery, and user behavior data to pool together to create a comprehensive profile of the household. Such personal sensitive data if accessed unauthorized can result in a privacy breach and the breach of sensitive information, thus such kind of data can only be very properly encrypted as well as have a user consent mechanism. Data security poses another critical issue. The risk of cyberattacks against the HDT system increases since continuous data passing between the physical house and its digital counterpart is required. Because malicious actors could exploit vulnerabilities in the framework to disrupt household systems or extract confidential information, one should run secure communication protocols and conduct regular audits of the system. In addition, compliance with legal regulations such as the General Data Protection Regulation (GDPR) or equivalent regulatory laws makes it even more complicated. While the modularity of the HDT is beneficial from a perspective of scalability and functionality, this can be problematic if all the components of the HDT do not satisfy regulatory requirements. One such application domain is collaborative filtering based on data of neighboring houses, and therefore they must be aware of cross-household consent and possibly avoid infringements of individual privacy rights. Moreover, there are ethical dilemmas in relation to autonomy and the making of decisions. With changing prescriptive and autonomous functionalities, we need to carefully manage the balance between user control and autonomous user actions. It also includes dealing with algorithmic transparency bias, and ensuring that decisions correspond to the user’s preferences and wellbeing. Additional development of HDTs must be based on ethical design principles and legal compliance frameworks such as privacy by designs, robust security measures, and pre-emptive involvement with the regulatory organs to build trust and protect users in IoT-enabled homes.

#### Case study 2: virtual smart home simulator for human activity recognition

The second case study brings about an application of digital twin (DT) technology to run smart home environments for the betterment of the Human Activity Recognition (HAR) algorithms [34]. The work presents, the simulated data using sensors in the VirtualHome environment integrated with the VirtualSmartHome simulator. The simulator conducts such behavioural modelling of the resident through avatars that reflect how real smart apartments would behave and generate synthetic data similar to real-world data collected from volunteers. Further, it was shown that this synthetic data is able to bridge a gap (between training and real-world applications) to an average of 80 % F1 score on real datasets when used to train HAR algorithms. From future directions, like growing datasets, enabling new smart homes, having advanced scenarios using multiple agents, and sensing new modalities, such as audio and radar sensors, the study concludes. These improvements are aimed at bringing an increased level of realism for data as well as expanding the HAR applications which leads to the development of more efficient smart home systems. This case study illustrates how DT technology can overcome the data sparsity challenges for the development of robust HAR solutions, and for extending smart home systems.

We identify several ethical and legal challenges related to synthetic data generation and application in smart home environments generated through the application of the VirtualSmartHome simulator in generating Human Activity Recognition (HAR). A privacy issue is a reason, because synthetic datasets, though anonymized, are based on real-world behavior patterns of the volunteers. Avatar-based simulations of this kind could be exploited for unintended sensitivity, which inadvertently compromises privacy. To address these concerns, it is essential to ensure strict anonymization protocols and get explicit consent for data usage. Another challenge is in securing data as synthetic datasets are increasingly used in collaborative environments or shared for research purposes. Data manipulation or exploitation from these datasets or from the simulator’s models is possible if unauthorized access is allowed. It shows that such data-sharing platforms need to be secure, access controls need to be in place and synthetic data repositories should be audited frequently. There are two things to verify from the regulatory compliance point of view, the first is that using synthetic data to train the HAR algorithms must be in accordance with the legal frameworks like GDPR and other privacy legislations. While synthetic data is not as sensitive as real-world data, the generation and use of synthetic data must be adhered to the rule of transparency and data protection. Next, for instance, the simulator has to explain how synthetic behaviors are close in quality to real-world ones in order for such use to be ethical.

These concerns are magnified when advanced sensing modalities such as audio and radar sensors are integrated. Even if audio is synthetic, it may cause privacy alarms since it similarly resembles real human speech. Powerful, radar data however also needs to be apprehensive of potential use cases involving such as unauthorized tracking of movement patterns. There also are ethical dilemmas inherent in forming complex multi-agent scenarios which simulate the interactions of residents. There could be scenarios in which these HAR algorithms reproduce biases or stereotypes unintentionally, leading to unfairness and inaccessibility of the algorithms. There must be the implementation of diversity in the behavior modeling and rigorous testing of the bias. Future developments in this domain need to focus on developing ethical data generation practices, safe data handling, and transparent reporting of methodology. If these challenges are addressed, VirtualSmartHome will be able to responsibly drive innovation in HAR solutions for smart homes.

#### Case study 3: enhancing smart homes with digital twin technology

The third case study focuses on the transformative nature of digital twin (DT) technology in improving smart home applications’ efficiency and user-friendliness [35]. First, the study presents the challenges existing in traditional smart home automation systems and describes the digital twins as a contemporary IoT framework advancement. The research presents the foundational concepts of digital twins and outlines the practical implementation of using digital twins in smart homes along with an experimental setup that compares the digital twin-based IoT systems with existing IoT systems. Results show that digital twins are a great way to greatly increase system efficiency by simulating IoT ideas and addressing common problems of smart home automation. A digital twin scenario could, for example, involve real-time responses such as capturing and relaying real-time photographs of humans or intruders to a camera attached to a microcontroller to improve security in the owner’s home. The presence of this advanced functionality shows that digital twins are a major addition to IoT architecture, providing personalized, context-aware solutions and more reliable smart home systems.

The employment of digital twin (DT) technology in smart home contexts brings forth new ethical and law issues that have to be considered to enable its use in a responsible manner. After all, digital twins are based on real-time data collected from IoT devices, and thus there are concerns regarding privacy. Monitoring of occupants’ behavior, movements, and interactions increases the risk of intrusive surveillance. Despite anonymization protocols, it is still possible to reidentify people through behavioral patterns, necessitating stringent protection for user privacy. The next critical challenge is to secure the data because digital twins require the transfer of large amounts of data between physical devices and their virtual counterparts. In the case of the DT ecosystem being susceptible to cyberattacks, sensitive information could be compromised, or the malicious actors could even gain the opportunity to manipulate home systems by changing the security settings or disabling critical functions. The integrity of data needs to be fortified through robust encryptions, regular software updates, and intrusion detection systems.

From a regulatory compliance point of view, one needs to ensure that you are meeting the requirements based on international privacy laws such as GDPR, HIPAA (if you are processing medical data), or country-specific IoT regulations. For real-time intruder detection and reporting, cameras and microcontrollers must be used in a way compliant with legal standards in the surveillance and sharing of data. For example, homeowners are to be informed as to how their data is processed, stored, and possibly shared with third parties. Moreover, there are ethical problems with users’ consent and autonomy. Data collection of a smart home needs to be well-informed for smart home occupants, as well as must be allowed to limit/control their contribution to the DT ecosystem. Enabling users requires transparent policies for building trust in the technology. Finally, there are a couple of challenges related to bias and inclusivity. There is a danger that design and implementation biases present in DT systems may accidentally reproduce biases of their design or implementation focusing on some demographic groups or ignoring accessibility needs. The key to maintaining fairness and equity is to ensure that DT-enabled smart home solutions are designed inclusively and tested by various user bases.

#### Case study 4: digital twin-driven smart home for assistive living

In this study, the potential of digital twin technology to enhance smart home systems is explored, targeting supporting elderly or frail individuals’ daily activities in collaboration with mobile assistive robots. The concept is based on digital twins to represent in detail the environment of the human user in order to develop personalized assistive solutions addressing the user’s individual needs [36]. It proposes a robot navigation and environment semantics integrated into open-source software as a proof of concept for a "digital twin-driven smart home." The implementation results so far show very promising initial insights into the building of such digital twins, and the practical applicability of the description to create smart spaces. This work details the differences in performance in this scenario between the actual and simulated environments and the benefits of digital twins in improving adaptability and functionality in smart homes. The system promotes a smooth coexistence between physical and virtual environments by utilizing digital twins which allows the assistive robots to navigate and understand their environment efficiently. This integration makes sure that assistive solutions are adaptable to the changing and dynamic requirements of the users. The key point is not that digital twins will let older people live in better smart home ecosystems, but that by using digital twins, we can lay the basis for raw, efficient, accessible smart home ecosystems that will improve people's lifestyles.

However, the implementation of digital twin (DT) technology to support assistive living raises several ethical and legal issues that must be burdened correspondingly to prevent the unmannerly use of this innovation. Given the need for comprehensive data about a user’s home environment, behavior, and routines, the user’s privacy concerns take precedence. As a result of continuous monitoring, creates hurdles regarding the intrusive quality of such monitoring and the risk of leaking sensitive personal information in case assistive robots are incorporated into the machine. In order to maintain trust in such systems, user anonymity and data exposure are essential to be maintained. In this context, data security is paramount as breaches can affect not only personal data but also the operational invocation of assistive robots and other connected devices. This could lead to undermining the safety of users who are vulnerable, because of violent cyberattacks, which would disrupt essential services, such as navigation and communication between the virtual and physical environments. Without them, comprehensive measures would be needed to secure the communication protocols in place and to detect real-time intrusions. Adding regulatory compliance is another layer of complexity when it comes to handling health-related data of elderly or frail individuals. By following international standards, such as HIPAA for handling health data and GDPR for wider data privacy rules, user data is handled ethically from a business perspective. In addition, the deployment of assistive robots should be compatible with national safety regulations and the certification of a robotic system for domestic use.

Informed consent can comprise an ethical consideration and user autonomy. For elderly and cognitively impaired people, understanding the scope of data collection and use could become challenging too. It is essential to implement a consent mechanism that is clear and can be accessed by all and the option for users to change or remove their membership from the ecosystem of DT. Additionally, assistive robots must integrate equity and inclusivity in their designs so that a wide variety of physical, cultural, and linguistic needs are taken into consideration. The system design can discretize the problem to such an extent that some groups are unintentionally being made to advantage. Balancing the independents and the dependents is finally a subtle job. Assistive technology could lead to over-reliance that leads to stakeholders being out of control, or lack of support that may lead to less safe and comfortable users. The key is finding that balance that allows you to strike the balance of what individuals need and prefer. This article is entrusted with addressing these ethical and legal challenges to make full use of the potential of digital twin-driven smart homes for assistive living to maintain quality of life while safely protecting the rights and dignity of users.

#### Case study 5: natural language processing-driven activity recognition in smart homes

In this study, we study an innovative approach to activity recognition in smart homes by combining methods from Time Series Classification (TSC) and Natural Language Processing (NLP) [37]. Activity recognition is a fundamental piece of smart home systems designed to provide automated services in accordance with the desired needs of the inhabitants. Nevertheless, challenges in the variability of environments, sensory-motor systems, user habits, sparsity of signals, and model redundancy hamper such recognition. To face this, the proposed method uses an embedding technique based on term frequency encoding in order to improve feature extraction and supposes domain knowledge to improve classification accuracy. Finally, it evaluates its method on two datasets obtained from the Center for Advanced Studies in Adaptive Systems (CASAS). Fully Convolutional Networks (FCNs) from TSC that are applied for the first time in this context are compared to Long Short-Term Memory (LSTM) networks to see their classification performance. Results show that FCNs outperform LSTMs in offline activity classification, and finally demonstrate the suitability of FCNs for offline smart home applications. Furthermore, event encoding and embedding significantly improve classifier performance, indicating the importance of domain knowledge when overcoming the limitations of end-to-end systems. Speaking generally, this is a very important advancement in activity recognition, providing a solid structure for the extraction of important features and introducing flexibility and trust in smart home automation systems.

A major ethical and legal concern is presented by the integration of Natural Language Processing (NLP) and Time Series classification (TSC) methods for activity recognition in smart homes. These are important problems to solve in order to develop and deploy responsible smart home systems. The major ethical issue is concerned with privacy. There has been extensive work on activity recognition systems that rely on big successive monitoring of user behaviors for which abundant sensitive data on daily routines and how people interact is collected. Such detailed data can be used to expose users to the risks of surveillance and misuse [38]. To guarantee protection of user privacy measures need to be taken for anonymizing and minimizing the data collection. Another challenge is data security, where putting elements into the vehicle poses a threat of data breaches, the more likely in our case if we resorted to embedding techniques and real-time data analytics. Unwonted exploitation or harm to users may in fact occur if malicious actors gain access to this data. However, since security is a fundamental aspect of smart home systems, state-of-the-art encryption protocols, secure communication channels, as well as robust authentication mechanisms need to be employed to prevent unauthorized access [39]. Different aspects of ethical concern in algorithmic processing include fairness and bias. Since the training datasets may contain biases of various kinds, the NLP models and classifiers might advance them to an unintended extent, resulting in unequal performance for different user demographics. To mitigate these biases and provide access to equitable outcomes for all users, during development, it is essential to ensure your future datasets are diverse and representative.

They need to be compliant with those regulatory frameworks, for example, GDPR which is a regulatory framework for the privacy and protection of the data. So that such regulations are met, the system’s data collection, storage, and processing methods must be carried out by these regulations to avoid legal complications and lead to user trust. Ethical imperatives include transparency and explainability. The activity recognition models need to be understandable by users as to how they create activity recognition models, what data is used by the models, and what decisions about creating activity recognition models are made. It’s also important to offer clear explanations about the functionality and limitations of these systems so users feel confident using these systems and as a way to indicate that consent is well-informed. Given the deployment of activity recognition systems in shared or multi-user environments, it is very important to have informed consent. The data collection and use practices must be aware of and agreed upon by all the inhabitants of the smart home [40]. The last of these is that automation in general may lead to overreliance. First, activity recognition enhances the functionality of smart home systems, but excessive dependency may deprive people of their independence. Awakening human capabilities is the aim behind automation with a striking balance between automation and manual control to achieve. The ethical and legal challenges of adoption of NLP-based activity recognition for smart homes have to be addressed to successfully leverage these innovations and they must be in line with societal values and user expectations.

### Technical mitigation strategies

The challenges posed by IoT in areas such as privacy, security, and legal compliance have prompted proactive measures from governments and industries worldwide. In India, significant initiatives have been launched to address these issues while promoting IoT adoption across various domains.

#### Industry and government initiatives to address challenges

**Fig. 5:**
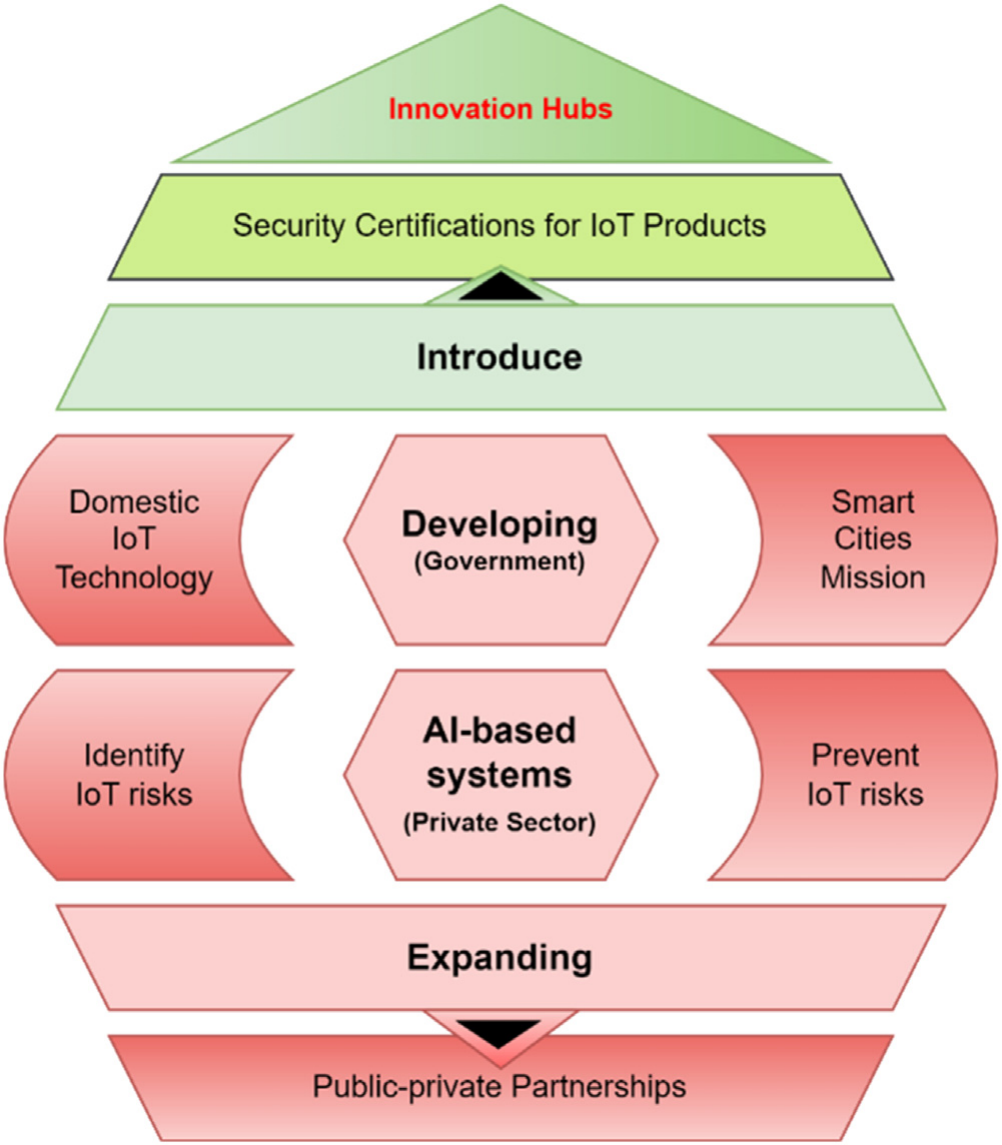
Industry and government initiatives to address challenges.

Fig. 5 represents how industry and government initiatives address these challenges. The Digital India program of the Indian government will be a major driver for IoT deployment, particularly in areas such as smart cities, healthcare, and agriculture. The program focuses on developing domestic IoT technology, reducing the dependency on foreign systems, and ensuring data security [41]. Additionally, the Smart Cities Mission has been incorporating IoT tools to improve urban management with a mandate for open data-sharing protocols for citizens' privacy. It is with that in mind, that these measures underpin the Government's dedication to establishing robust IoT frameworks. The recently proposed Digital Personal Data Protection (DPDP) Bill, 2023 in addition to the existing legal framework puts India on a pedestal to confront IoT-specific legal challenges head-on. It has an emphasis on user consent, secure data management, and rights for data subjects to this effect. It creates an outline in which businesses can place their IoT devices because they will meet strict privacy standards. In addition, the National Cyber Security Strategy strengthens cybersecurity measures & protocols by planning in advance so that IoT ecosystems are designed and implemented with cyber security parameters.

The private sector has also played a big role, partnering with academia and global organizations to establish Security standards aligned with Indian infrastructure. Companies within the industry are exploring AI-based systems to identify and prevent IoT risks like unauthorized access or an encrypted data leak [42]. With an increasing demand for experts capable of overseeing and harnessing the IoT, training programs in cybersecurity are being launched [39]. Potential outcomes include expanding public-private partnerships, developing innovation hubs, and introducing security certifications for IoT products. User campaigns to be incorporated as part of public awareness about safe IOT practices i.e. Your password must be something different, enable the MFA whatever you have. The concentrated efforts of India to address new-age innovation with a firm data-protection regime are illustrative of an evolutionary and proactive adoption of the IoT ecosystem. India has become a model for safe and ethical integration by improving policy, collaboration, and embracing User Rights.

#### Best practices for ethical IoT development

One positive element of the Network Service Millennium is realizing that an ethical IoT development does not only demand handling privacy and security measures but it should also suit inclusivity in sustainability form. The basic thing is to get the data in a clear and consented (by user) way, and work on it [43]. Only basic data should be collected, and the usage of it must be transparent to users, complying with regulations like GDPR or India's DPDP Bill. To avoid unauthorized access as well as data breaches, strong security measures like encryption, authentication, and regular updates are required [44]. Needless to say, IoT systems should also explain their black-box functionalities and share the system transparency as well as shedding light on how a decision was made, where splits accountability framework between manufacturers and users alike.

Table 6 outlines best practices for ethical IoT development, emphasizing critical aspects like data privacy, user autonomy, and regulatory compliance to address ethical and legal challenges effectively [45]. Ethical IoT Requires Inclusivity and Accessibility Developers should take care to design interfaces and functionalities that work for a high fraction of the human population, including the disabled, or only having limited levels of digital literacy. Sustainability is another huge factor that involves the implementation of energy-efficient designs, eco-friendly materials, and decreasing e-waste by recycling drives. Equity and the prevention of bias are crucial for AI-powered systems. This involves checking algorithms and employing varied datasets to steer clear of biased results [[46], [47], [48]]. Allowing users to personalize their settings, control over-ride functions, consent for human data review and you enhance trust and give people a sense of reduced risk in taking back some measure of control over their devices and data. And, crucially, collaborative efforts of stakeholders—across governments, industries, and academia—are necessary to meet evolving challenges and continuously improve ethical standards. These arm's-length relationships can facilitate a strong framework that balances innovation with user rights and security enabled [49]. When developed ethically, IoT has a cohesive future as enabling secure, inclusive, and safe paths towards more efficiency — however, being ethical could mean opening the development of an IoT product to continuously consider broader base surface attacks than just compliance only.

**Table 6:** Best practices for ethical IoT development.

Practice	Description	Objective	Example
Privacy by Design	Embedding privacy into the design and architecture of IoT systems from the outset.	Protect user data and minimize risks of breaches.	Designing a smart thermostat that anonymizes and encrypts user data before transmission.
Transparency	Providing clear and concise information about data collection, storage, and sharing practices.	Build user trust and ensure informed consent.	Displaying data usage policies directly in a device's mobile app interface.
User-Centric Design	Allowing users to customize and control their privacy settings easily.	Empower users to manage their own data.	Enabling users to opt in or out of specific data-sharing features on a connected fitness device.
Secure Communication	Ensuring robust encryption and secure protocols for data transmission between IoT devices.	Prevent unauthorized access and data breaches.	Using end-to-end encryption for video feeds in smart home security cameras.
Regular Audits and Updates	Conducting frequent security audits and providing firmware updates to address vulnerabilities.	Maintain system integrity and adapt to new threats.	Issuing automatic software updates for IoT devices to fix identified security flaws.
Minimal Data Collection	Collecting only data that is absolutely necessary for device functionality.	Reduce exposure to data misuse and privacy risks.	A smart speaker that processes voice commands locally without storing them on external servers.
Anonymization of Data	Implementing techniques to remove or obscure personally identifiable information in collected data.	Safeguard user identities in case of breaches.	Masking user-specific identifiers in datasets shared with third-party analytics services.

Table 7 assesses the effectiveness of different best practices in ethical IoT development with regard to privacy, security, and openness. Data minimization can be said to exemplify best practices for enhancing privacy as well as dampening security risks and increasing transparency where unnecessary information is avoided. Further, end-to-end encryption, which is usually considered to provide an ultimate security solution, reacts negatively to transparency since users won’t understand how their data is processed [[50], [51], [52]]. Security activities increase security but do not look at privacy or at providing more transparent systems. The means of the User’s consent lend some support to the principles of privacy and transparency; however, they do not necessarily strengthen security. Legal requirements fully work for privacy, security, and transparency and thus can be viewed as a holistic best practice for ethical IoT. These practices explain how privacy and security should be harmonized with transparency as IoT technology continues to advance in order to sustainably grow.

**Table 7:** Effectiveness of different best practices in ethical IoT development.

Best Practice	Enhances Privacy	Improves Security	Ensures Transparency
Data Minimization	✓	✓	✓
End-to-End Encryption	✓	✓	✗
Regular Security Audits	✗	✓	✗
User Consent Mechanisms	✓	✗	✓
Compliance with Legal Frameworks	✓	✓	✓

✓ = Positive Impact (Supports Better Management), ✗ = Negative/Neutral Impact (Requires Additional Focus)

#### Legal frameworks for better compliance

At the same time, legal frameworks are important to provide stronger incentivization of good behaviors in IoT development and usage — particularly as these devices come to hold more sensitive data and affect various critical infrastructures. These frameworks need to be robust and address privacy, security, and accountability by providing developers, manufacturers, and users with clear guidelines. Laws such as the General Data Protection Regulation (GDPR) in Europe and the California Consumer Privacy Act (CCPA) ushered in new standards for responsible data protection, detailing user approval, transparency, and access/removal rights of personal data. These laws ensure stiff penalties for non-compliance, which motivates organizations to implement and follow secure and ethical practices. The Digital Personal Data Protection Bill (DPDP) in India is seen as an appropriate legislation to define a customized IoT regulatory framework. They are centered on the protection of individual data rights; the definition and specification of easily understood and well-publicized data processing obligations where that is legally justified, and justifying lawful data transfer [53]. Legal frameworks must address emerging IoT-specific concerns, including cross-border data flow and liability for system failures and cybersecurity breaches. Instead, these frameworks answer this existential question "Who is responsible", hold manufacturers, service providers, and third-party entities accountable so that the entire ecosystem is balanced ensuring user protection. Recent global initiatives like the ISO/IEC standards essential for IoT security and privacy are set to be a supporting technical standardization under the new legislation. Governments are also investigating IoT applications in healthcare, transportation and smart homes for sector-specific regulations due to the different risks involved [54]. Agreeing on legal frameworks across borders is essential to help legitimize large-scale global deployments of IoT solutions, adhering to local customs and tastes in the process. In this way, legal frameworks can increase compliance and support responsible innovation within IoT systems.

#### Cybersecurity standards and safeguards for home digital twins

To tackle the particular security issues of cybercrime that emerge when a Home Digital Twin is in use, strong safeguards and adherence to existing standards are required. Standards such as ETSI EN 303 645 and NIST IoT Cybersecurity Guidelines provide minimum benchmarks encompassing aspects like data protection, strong authentication mechanisms, secure software updates, etc. For example, Home Digital Twins can use end-to-end encryption such as TLS 1.3 to ensure the safety of the data being exchanged. By doing this, no other device can tamper communication between two devices, including user information. MFA and strong password management are an additional layer of security enhancements. Real-time notifications about access or activities that are not suspected help users keep controlling their digital environments both actively and being able to watch everything that is taking place automatically. Additionally, good key management systems can also improve the reliability and integrity of devices’ authentication processes. In line with the GDPR and DPDP, data protection strategies such as minimization and anonymization are vital to align with. With Home Digital Twins you can collect a minimal amount of data and be safely anonymous. When dealing with aggregate datasets, adding a layer of security using privacy-enhancing techniques like differential privacy can be a good idea.

Proactive defense is offered through advanced cybersecurity measures like AI and machine learning models that integrate. Continuous monitoring of AI-driven device activity can be done continuously to identify abnormal behavior patterns that indicate such cyber-attacks may have been launched. Intrusion detection systems (IDS) can react in real-time against the threats thereby reducing its risk of being compromised. Frequent updates of the firmware, and frequent security audits in the 3rd party back-end are essential. This will also contribute towards increasing the security of IoT devices as manufacturers are held accountable and worked with to develop specialized certification programs for their devices. Also, user education campaigns are essential. Raising awareness on best practices like letting MFA, not using weak passwords, and detecting phishing is one of the areas in these campaigns that should be addressed. A role is also that of bringing such user-friendly interfaces that will assist users in carrying out their secured configurations so that it might result in the mass adoption of security protocols. With these measures in place, Home Digital Twins can address cybersecurity challenges as a whole, offering users confidence in use and operation reliability. By way of such efforts, an ecosystem with secure and resilient ecosystem, where privacy and innovation live together smoothly, is promoted.

Strong cybersecurity standards are necessary to address security vulnerabilities and reduce risks in the rapidly developing ecosystem of IoT. The comparison of prevalent security standards is presented in Table 8, which compares the adoption rate, ability to reduce breaches, and major focus areas for each of the standards that they’ve adopted. By making this table, points to the growing importance of aligning security practices with well-formulated frameworks to make robust and secure IoT systems among practitioners in industry and even researchers. However, ISO/IEC 27001 has a high adoption rate of 67 %, largely because of its comprehensive, risk management and data security approach. It gives a systematic framework for the management of sensitive information, data confidentiality, and the reduction of risks in many other sectors. ISO/IEC 27001 has a breach reduction effectiveness value of 78 %, which underlines its importance in protecting organizational security.

**Table 8:** Comparative effectiveness of cybersecurity standards in IoT systems.

Standard	Adoption Rate (%)	Effectiveness in Reducing Breaches (%)	Primary Focus
ISO/IEC 27001	67	78	Risk management and data security
NIST Cybersecurity Framework	53	85	Identification and mitigation
ETSI EN 303 645	42	70	IoT-specific security measures
Zero Trust Architecture (ZTA)	30	90	Continuous verification

**Fig. 6:**
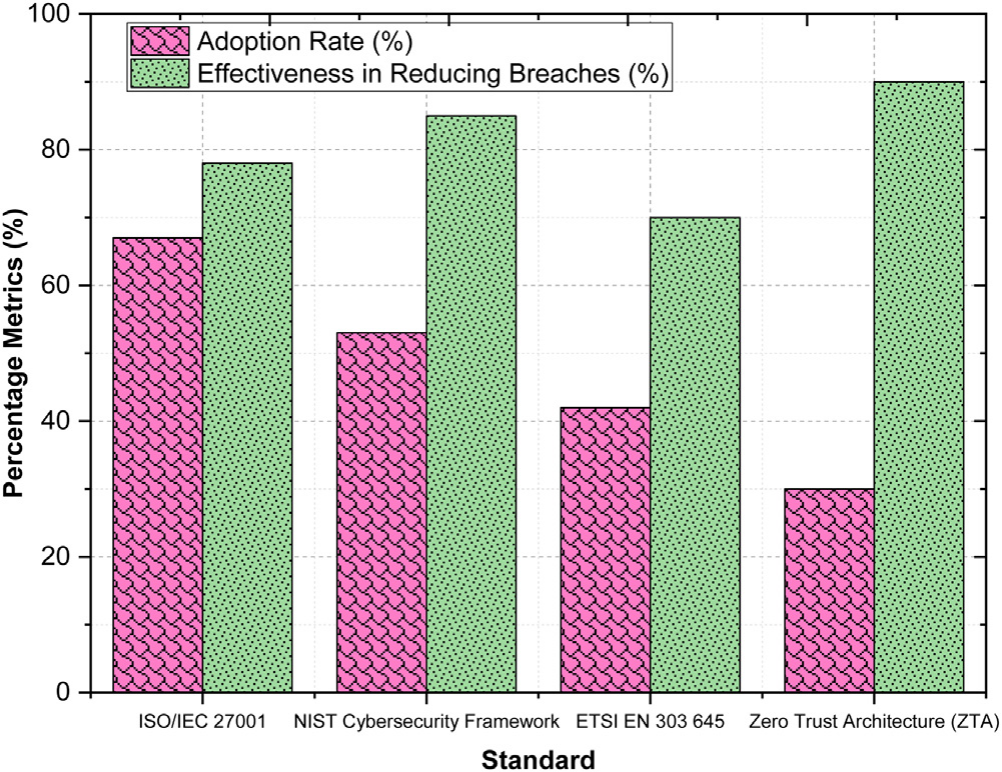
Comparative effectiveness of cybersecurity standards in IoT systems.

The NIST Cybersecurity Framework is impressive in its ability to reduce breaches and flip that number to an 85 % level as shown in Fig. 6. The framework has a 53 % adoption rate and it is widely acknowledged for the ability to identify, mitigate, and respond to threats. It is therefore an important tool for organizations who are targeting the development of a strong cybersecurity posture. It is the ETSI EN 303 645 standard produced to cater to IoT environments with an adoption rate of 42 % and breach reduction effectiveness of 70 %. Its focus on IoT-specific security measures, such as secure device communication and default password management, highlights its relevance in the context of connected devices. Of all the models, Zero Trust Architecture still has the lowest adoption rate of 30 %, but it is the most effective model in preventing breaches with 90 %. This standard also makes explicit continuous verification and is premised on no single entity being inherently trusted, internal or external. Most of the vendors either have no proactive approach toward threat management or their threat management approach is reactive. Finally, Table 8 shows the different rates of adoption and effectiveness of cybersecurity standards as well as reveals the necessity of adopting cybersecurity standards where businesses find them. Ensuring the security and sustainability of IoT ecosystems requires a balanced approach to adoption feasibility and effectiveness.

#### Proposed guidelines for ethical and legal challenges in IoT

1. **Strengthening Data Privacy Measures:** Data in IoT systems should be protected against potential data breaches by leveraging strong encryption algorithms, such as AES (Advanced Encryption Standard) or RSA (Rivest Shamir Adleman), to secure data when it is in transit or at rest. Data breaches or potential security lapses are easily identified and resolved with regular audits and vulnerability assessments. It is essential to implement a framework to protect the privacy of individuals while still deriving value from data analysis be it analytical, exploratory or statistical modeling. This compliance goes hand in hand with building user confidence, and even trust, in IoT systems.
2. **Implementing Regulatory Compliance Mechanisms:** Organizations should determine the legal framework that governs their practices like GDPR, CCPA, or the India DPDP Bill and map their practices to those frameworks. This can include creating a compliance checklist and designating DPOs (Data Protection Officers) to help ensure the jurisdictions are being adhered to. Moreover, organizations must monitor changing legal requirements and update their policies to meet new forms of regulation, such as AI regulation. To mitigate the risks, strategically engage top-tier legal experts and consultants to ensure that your product offering is in complete alignment with the local regulatory environment.
3. **Enhancing User Awareness and Control:** Designing dashboards that are user-centric, where people can view, manage and delete their data, is critical for transparency. Educational campaigns, like webinars or interactive tutorials, enable users to make informed decisions regarding the utilization of their data. This requires the implementation of multi-factor authentication (MFA) and fine-grained permission controls, enabling users to manage who can access their IoT devices. Such initiatives are targeted at ensuring that users are active participants in the protection of their privacy and security, instead of passive recipients of technology.
4. **Developing Industry Standards and Certifications:** The standardization of a secure IoT device and data practices helps to minimize differences between manufacturers. Best practice is encouraged by certification programs — like ISO/IEC 27001 for information security. This can help consumers and companies have standards that are recognized for all IoT devices, to really spot the ones that are more secure and ethical. Such industry collaboration can further help accelerate the development of significant standards.
5. **Proactive Cybersecurity Practices:** Its utmost imperative to fully understand the risks that a particular system might face on a consistent basis and perform the exercise to identify vulnerabilities in a proactive way. Manufacturers must embrace a “secure-by-design” strategy, incorporating security at every stage of the device lifecycle. Conducting threat modelling and regular penetration tests can find weaknesses before attackers do. Establishing incident response teams will quickly address breaches, preventing them from causing more damage. This holistic approach is crucial to ensuring the security and reliability of an IoT ecosystem.
6. **Fostering Ethical Design Principles:** Ethical consideration should not only be limited to functionality but also to equity, inclusivity, and transparency. Consider for example that, IoT systems that leverage AI should be audited for the presence of bias to ensure equitable outcomes for all users. Mitigating such misrepresentation and misuse comes down to open communication regarding risk and limitations of IoT devices. Thus, while innovation drives product development cycles, ethical practices ensure that these innovations remain beneficial to society, thereby fostering a sustainable pace of IoT development.

#### Ethical accountability in practice

Ethical accountability in IoT systems focus on user empowerment, transparency and moral alignment at the design and deployment phase. These practical measures show examples of actions that can implement ethical accountability:

**1. User Control over Personal Data:**

○ Communicate easily with interfaces for users to access their data and options to view, edit, delete, or transfer their data.
○ Implement granular privacy controls, which allow users to customize permissions for sharing of data on a case-by-case basis.
○ Utilize consent dashboards and similar tools to guarantee that users can access and manage their consent at any given moment and to communicate relevant updates of their data usage.

**2. Transparency in Data Processing:**

○ Provide interactive dashboards for real-time visibility into data processing, storage, and sharing of data.
○ Use plain language in privacy policies and terms of service to ensure users understand how complex IoT is working.
○ Use transparency tools such as AI explainability modules that provide insights into how IoT systems make their decisions.

**3. Ethical IoT Design:**

○ Implement the principle of “privacy by design” and Embed privacy protections throughout every step of the system development lifecycle.
○ Perform impact assessments for ethical risks and benefits prior to deploying IoT devices in homes.
○ Promote inclusivity by ensuring that IoT interactions are usable for people with disabilities, or limited digital experience.

**4. Accountability Mechanisms:**

○ Implement transparent reporting channels for security or privacy concerns, supported by responsive remediation processes.
○ Implement comprehensive audit trails capturing access and data modifications, promoting comprehensive accountability of the actors within the system.
○ Establish and articulate common responsibilities of manufacturers, service providers, and users in protecting the ecosystem.

**5. User Empowerment Through Education:**

○ Conduct awareness campaigns for users regarding safe IoT practices (enabling MFA, keeping strong passwords, etc).
○ Provide hands-on tutorials and resources to explain IoT functionalities to demystify it and encourage active participation from end users in ethical system usage.

Implementing such measures would allow IoT systems to embrace ethical accountability, especially for Home Digital Twins, engendering trust, and paving the path for balancing innovation and rights.

#### Industry best practices

In the market of IoT, industries leading the IoT space have also adopted several new-age practices to address the challenges of ethical and security of IoT systems. Few notable examples include:

**Privacy by Design:** Security in the Cloud Cloud-based IoT devices have some advantages, but companies still need to be proactive about privacy and data protection. It would encompass encrypting information on the client side while stored and transferred, performing whatever manipulation possible locally to resident devices vs over the wire to servers maintained away from home, and providing transparent choices to user on how much privacy they are willing to give up. For instance, Apple’s HomeKit platform — which enables deep integration through sharing information across IoT devices — prioritizes security in the way data is structured and transferred to devices.

**Adoption of Secure Development Lifecycles (SDL):** Building on these experiences, Microsoft’s Security Development Lifecycle (SDL) brings security to the forefront as a fundamental part of the IoT product development process, starting at design and continuing through updates after deployment. This top-down model encompasses threat modelling, extensive code scrutiny, vulnerability assessments, and secure default settings. As a result of this early-stage security embedding process, Microsoft dramatically reduces the number of potential vulnerabilities while ensuring system services remain up and running, guaranteeing the availability and reliability of critical products. SDL has set the standard for IoT developers looking to produce resilient products that are immune to new cybersecurity risks.

**Decentralized Identity Management:** IBM also uses blockchain to decentralize identity management in IoT networks. Parts of those credentials are verifiable, without relying on centralized sources, giving users control over their own data. Because of the immutability and transparency of Blockchain, these addresses secured authentication and data integrity and grants that unauthorized personnel cannot get access or tamper with the data. Decentralized identity systems provide more secure, private, and efficient management for IoT devices, which is critical for the development of trust and accountability in IoT ecosystems (including in sensitive applications such as healthcare and connected homes), evidenced by IBM's work on the Hyperledger Indy project.

**AI-Driven Threat Detection:** Amazon uses sophisticated AI and machine learning models to get near real-time alerts of potential security threats across its IoT devices. The systems analyze patterns of behavior from devices to identify anomalous behavior, such as unauthorized access attempts or unusual data flows, before they become problematic. Such insights powered by AI help facilitate automated responses — e.g., quarantining infected devices or notifying users. One such system is leveraged in Amazon Ring security devices to protect user privacy and user access to functionality in smart home environments.

**Cross-Industry Collaboration:** Organisations such as the IoT Security Foundation (IoTSF) are working collaboratively to identify common best practice across sectors with the aim of strengthening security across the Internet of Things. These include initiatives to provide a framework for safe deployment of IoT devices, tackling vulnerabilities, and information sharing. IoTSF’s frameworks promote the prioritization of security certifications, promotion of ethical data practices, and implementation of strong cybersecurity for manufacturers. Cross-industry collaborations bring together stakeholders to ensure solutions are innovative, secure, and trustworthy, giving rise to the right IoT solutions.

## Conclusion

The rapid proliferation of IoT devices, particularly in Home Digital Twins, has transformed modern living with enhanced convenience, efficiency, and personalization. However, these advancements are accompanied by critical ethical and legal challenges, including privacy breaches, data security vulnerabilities, and regulatory complexities. This study underscores the need to balance innovation with ethical accountability, emphasizing transparent, user-centric design, and robust security measures. Key research findings highlight the importance of embedding ethical accountability throughout the IoT lifecycle, incorporating practices such as privacy-by-design, strong encryption, and user empowerment mechanisms. Decentralized architectures, such as blockchain, can bolster transparency and accountability, while AI-driven threat detection offers dynamic responses to emerging risks. Furthermore, adopting zero-trust security models and harmonizing global standards like GDPR and ISO/IEC can enhance trust and compliance across jurisdictions. Actionable recommendations include strengthening regulatory frameworks, fostering public-private partnerships, and promoting collaboration among stakeholders—governments, industries, academia, and end-users. These efforts aim to build resilient and trustworthy IoT ecosystems, ensuring innovation aligns with societal values and fundamental rights.

Future IoT advancements must prioritize sustainability and inclusivity, including energy-efficient designs, eco-friendly materials, and accessibility for diverse user groups. Innovations such as adaptive IoT architectures can enable seamless integration with renewable energy systems, reducing environmental footprints. Collaborative frameworks among global stakeholders can accelerate the development of standardized protocols to address cross-border data governance challenges, ensuring interoperability and compliance. Decentralized data architectures, such as blockchain, can offer enhanced transparency, secure data transactions, and improved user control. Furthermore, advancements in AI and machine learning will empower IoT systems to anticipate user needs, optimize resource allocation, and proactively address vulnerabilities. Public awareness campaigns and educational initiatives are also essential to equip users with the knowledge and tools to engage responsibly with IoT systems. By integrating these strategies, IoT ecosystems can evolve into trusted, equitable, and resilient frameworks that align technological innovation with ethical principles and societal well-being. This holistic approach will transform Home Digital Twins into not only functional tools but also enablers of sustainable and inclusive progress.

## Ethics statements

Not applicable.

## CRediT authorship contribution statement

**D. Dhinakaran:** Conceptualization, Methodology, Writing – original draft. **S. Edwin Raja:** Visualization, Investigation, Software. **A. Ramathilagam:** Data curation, Software, Validation. **G. Vennila:** Methodology, Writing – review & editing. **A. Alagulakshmi:** Writing – review & editing, Investigation.

## Declaration of competing interest

The authors declare that they have no known competing financial interests or personal relationships that could have appeared to influence the work reported in this paper.

## References

[1] Bouchabou D., Grosset J., Nguyen S.M., Lohr C., Puig X. (2023). A smart home digital twin to support the recognition of activities of daily living. Sensors.

[2] Singla A. (2024). The evolving landscape of privacy law: balancing digital innovation and individual rights. Indian J. Law.

[3] Dhinakaran D., Ramani R., Edwin Raja S., Selvaraj D. (2025). Enhancing security in electronic health records using an adaptive feature-centric polynomial data security model with blockchain integration. Peer Peer Netw. Appl..

[4] Selvaraj D., Jeno J., Ramani R., Dhinakaran D., Prabaharan G. (2025). AFCP data security model for EHR data using blockchain. J. Cybersecur. Inf. Manag..

[5] Fakhouri H.N., AlSharaiah M.A., Al hwaitat A.K., Alkalaileh M., Dweikat F.F. (2024). Proceedings of the 2024 2nd International Conference on Cyber Resilience (ICCR), Dubai, United Arab Emirates.

[6] Altun C., Tavli B., Yanikomeroglu H. (2019). Liberalization of digital twins of iot-enabled home appliances via blockchains and absolute ownership rights. IEEE Commun. Mag..

[7] Grönman J., Rantanen P., Saari M. (2023). Proceedings of the 2023 IEEE 27th International Conference on Intelligent Engineering Systems (INES), Nairobi, Kenya.

[8] Dhinakaran D., Joe Prathap P.M (2022). Protection of data privacy from vulnerability using two-fish technique with Apriori algorithm in data mining. J. Supercomput..

[9] Shome P.P., Khan T., Kishk A.A., Antar Y.M.M. (2023). Quad-element MIMO antenna system using half-cut miniaturized UWB antenna for IoT-based smart home digital entertainment network. IEEe Internet Things J..

[10] Hemdan E.E., El-Shafai W., Sayed A. (2023). Integrating digital twins with iot-based blockchain: concept, architecture, challenges, and future scope. Wirel. Pers. Commun..

[11] Dhinakaran D., Selvaraj D., Dharini N., Raja S.E, Priya C.S.L. (2023). Towards a novel privacy-preserving distributed multiparty data outsourcing scheme for cloud computing with quantum key distribution. Int. J. Intell. Syst. Appl. Eng..

[12] Marco Bisanti G., Mainetti L., Montanaro T., Patrono L., Sergi I. (2023). Digital twins for aircraft maintenance and operation: a systematic literature review and an IoT-enabled modular architecture. Internet Things.

[13] Liu J., Li C., Bai J., Luo Y., Lv H., Lv Z. (2023). Security in IoT-enabled digital twins of maritime transportation systems. IEEE Trans. Intell. Transp. Syst..

[14] Macías A., Muñoz D., Navarro E., González P., Bravo J., Ochoa S., Favela J. (2023). Proceedings of the International Conference on Ubiquitous Computing & Ambient Intelligence (UCAmI 2022).

[15] Gupta D., Kayode O., Bhatt S., Gupta M., Tosun A.S. (2021). Proceedings of the 2021 IEEE 7th International Conference on Collaboration and Internet Computing (CIC), Atlanta, GA, USA.

[16] Padmapriya V., Srivenkatesh M. (2023). Digital twins for smart home gadget threat prediction using deep convolution neural network. Int. J. Adv. Comput. Sci. Appl. (IJACSA).

[17] Sartaj H., Ali S., Yue T., Moberg K. (2024). Model-based digital twins of medicine dispensers for healthcare IoT applications. Softw. Pract. Exp..

[18] Navigating the digital maze: privacy and security challenges in the era of cloud computing - Harsh Pandey, Chinmay Lunia, Er. Nisha Rathore - IJFMR Volume 6, Issue 3, May-June 2024. DOI 10.36948/ijfmr.2024.v06i03.23105

[19] Siddiqua Oosman B., Dudhe R. (2021). Proceedings of the 2021 International Conference on Computational Intelligence and Knowledge Economy (ICCIKE), Dubai, United Arab Emirates.

[20] Greser J. (2020). Proceedings of the ETHICOMP 2020. Paradigm Shifts in ICT Ethics, Logroño, Spain.

[21] Anwar S., Panda U., Mohapatra H. (2024). Legal and ethical issues in IoT based smart city: data privacy, surveillance, and citizen rights. J. Comput. Sci. Eng. Softw. Test..

[22] AboBakr, Azer M.A. (2017). Proceedings of the 2017 12th International Conference on Computer Engineering and Systems (ICCES), Cairo.

[23] Zeng Q., Pan Z., Zhang Q., Han T., Zheng W., Li J., Zhang Z., Feng W., Zhen Li, Ma L., Liu K., Yuan X., Xing S. (2024). CSR evolution: new opportunities and challenges for IoT in advancing ESG practices. Int. J. Front. Eng. Technol..

[24] Wachter S. (2018). Proceedings of the Living in the Internet of Things: Cybersecurity of the IoT - 2018, London.

[25] Zakerabasali S., Ayyoubzadeh S.M. (2022). Internet of Things and healthcare system: A systematic review of ethical issues. Health Sci. Rep..

[26] Ryan P.J., Watson RB. (2017). Research challenges for the Internet of Things: what role can or play?. Systems.

[27] D’Mello O., Gelin M., Khelil F.B., Surek R.E., Chi H., Doss R., Piramuthu S., Zhou W. (2018). Future Network Systems and Security. FNSS 2018. Communications in Computer and Information Science.

[28] Rajora R., Rajora A., Sharma B., Aggarwal P., Thapliyal S. (2024). Proceedings of the 2024 4th International Conference on Innovative Practices in Technology and Management (ICIPTM), Noida, India.

[29] Dhinakaran D., Joe Prathap P.M. (2022). Preserving data confidentiality in association rule mining using data share allocator algorithm. Intell. Autom. Soft Comput..

[30] Burd B., Elahi A., Russell I., Barker L., Pérez A.F., Siever B., Divitini M., Parker A., Tudor L., Guerra J.G. (2017). Proceedings of the 2017 ACM Conference on Innovation and Technology in Computer Science Education (ITiCSE '17).

[31] Falkowski P., Osiak T., Wilk J., Prokopiuk N., Leczkowski B., Pilat Z., Rzymkowski C. (2023). Study on the applicability of digital twins for home remote motor rehabilitation. Sensors.

[32] Dhinakaran D., Srinivasan L., Gopalakrishnan S., Anish T.P. (2025). An efficient data mining technique and privacy preservation model for healthcare data using improved darts game optimizer-based weighted deep neural network and hybrid encryption. Biomed. Signal. Process. Control.

[33] Elfarri E.M., Rasheed A., San O. (2023). Artificial intelligence-driven digital twin of a modern house demonstrated in virtual reality. IEEe Access.

[34] Bouchabou D., Grosset J., Nguyen S.M., Lohr C., Puig X. (2023). A smart home digital twin to support the recognition of activities of daily living. Sensors.

[35] Gopinath V., Srija A., Neethu Sravanthi C. (2018). Re-design of smart homes with digital twins. Journal of Physics: Conference Series.

[36] Asvadi A., Mitriakov A., Lohr C., Papadakis P., Aloulou H., Abdulrazak B., de Marassé-Enouf A., Mokhtari M. (2022). Participative Urban Health and Healthy Aging in the Age of AI. ICOST 2022. Lecture Notes in Computer Science.

[37] Bouchabou D., Nguyen S.M., Lohr C., LeDuc B., Kanellos I., Li X., Wu M., Chen Z., Zhang L. (2021). Deep Learning for Human Activity Recognition. DL-HAR 2021. Communications in Computer and Information Science.

[38] Lyu Z., Yang J.J., Lam M.S., Landay J.A. (2022). Proceedings of the 35th Annual ACM Symposium on User Interface Software and Technology (UIST '22 Adjunct).

[39] Dhinakaran D., Srinivasan L., Udhaya Sankar S.M., Selvaraj D. (2024). Quantum-based privacy-preserving techniques for secure and trustworthy internet of medical things an extensive analysis. Quantum Inf. Comput..

[40] Guittoum A., Aïssaoui F., Bolle S., Boyer F., Palma N.D. (2023). Proceedings of the 38th ACM/SIGAPP Symposium on Applied Computing (SAC '23).

[41] Selvaraj D., Udhaya Sankar S.M., Dhinakaran D., Anish T.P. (2023). Outsourced analysis of encrypted graphs in the cloud with privacy protection. SSRG Int. J. Electr. Electron. Eng..

[42] Dhinakaran D., Joe Prathap P.M, Selvaraj D., Arul Kumar D., Murugeshwari B. (2022). Mining privacy-preserving association rules based on parallel processing in cloud computing. Int. J. Eng. Trends Technol..

[43] Dhinakaran D., Joe Prathap P.M. (2022).

[44] Bugeja J., Jacobsson A., Davidsson P. (2022). The ethical smart home: perspectives and guidelines. IEEe Secur. Priv..

[45] Qayyum F., Alkanhel R., Muthanna A. (2023). Maximizing efficiency in energy trading operations through iot-integrated digital twins. Sensors.

[46] Salama F., Tsirkunenko A., Korkan E., Käbisch S., Steinhorst S. (2023). Proceedings of the 2023 IEEE International Conference on Omni-layer Intelligent Systems (COINS), Berlin, Germany.

[47] Sai Aswin B.G., Vishnubala S., Dhinakaran D., Kumar N.J., Udhaya Sankar S.M., Mohamed Al Faisal A.M. (2023). Proceedings of the 2023 World Conference on Communication & Computing (WCONF), RAIPUR, India.

[48] Zhu Q., Xu Z. (2020). Cross-Layer Design for Secure and Resilient Cyber-Physical Systems.

[49] Harini M., Dhinakaran D., Prabhu D., Udhaya Sankar S.M., Pooja V., Sruthi P.K (2023). Proceedings of the 2023 World Conference on Communication & Computing (WCONF), RAIPUR, India.

[50] Park K.T., Son Y.H., Noh S.D. (2021). The architectural framework of a cyber physical logistics system for digital-twin-based supply chain control. Int. J. Prod. Res..

[51] Saad A., Faddel S., Youssef T., Mohammed O.A. (2020). On the implementation of IoT-based digital twin for networked microgrids resiliency against cyber-attacks. IEEE Trans. Smart Grid.

[52] Banks R., Jones J., Hazzazi N., Garcia P., Zimmermann R. (2021). ITNG 2021 18th International Conference on Information Technology-New Generations.

[53] Lee J., Azamfar M., Singh J., Siahpour S. (2020). Integration of digital twin and deep learning in cyber-physical systems: towards smart manufacturing. IET Collab. Intell. Manuf..

[54] Ahmadi-Assalemi G., Al-Khateeb H., Maple C., Epiphaniou G., Alhaboby Z.A., Alkaabi S., Alhaboby D., Jahankhani H., Kendzierskyj S., Chelvachandran N., Ibarra J. (2020). Cyber Defence in the Age of AI, Smart Societies and Augmented Humanity.

